# Whole-Genome Identification and Expression Pattern of the Vicinal Oxygen Chelate Family in Rapeseed (*Brassica napus* L.)

**DOI:** 10.3389/fpls.2017.00745

**Published:** 2017-05-09

**Authors:** Yu Liang, Neng Wan, Zao Cheng, Yufeng Mo, Baolin Liu, Hui Liu, Nadia Raboanatahiry, Yongtai Yin, Maoteng Li

**Affiliations:** ^1^Department of Biotechnology, College of Life Science and Technology, Huazhong University of Science and TechnologyWuhan, China; ^2^Hubei Collaborative Innovation Center for the Characteristic Resources Exploitation of Dabie Mountains, Huanggang Normal UniversityHuanggang, China

**Keywords:** vicinal oxygen chelate proteins (VOC), *B. napus*, gene duplication, gene evolution, drought stress, gene expression

## Abstract

Vicinal oxygen chelate proteins (VOC) are members of the metalloenzyme superfamily, which plays roles in many biological reactions. Some members of the VOC superfamily have been systematically characterized but not in *Brassica napus*. In this study, 38 *VOC* genes were identified based on their conserved domains. The present results revealed that most of the *BnaVOC* genes have few introns, and all contained the typical VOC structure of βαβββ modules. The *BnaVOC* genes are distributed unevenly across 15 chromosomes in *B. napus* and occur as gene clusters on chromosomes C5 and A6. The synteny and phylogenetic analyses revealed that the *VOC* gene family is a consequence of mesopolyploidy events that occurred in *Brassica* evolution, and whole-genome duplication and segmental duplication played a major role in the expansion of the *BnaVOC* gene family. The expression profile analysis indicated that the expression of most *BnaVOC*s was increased in the leaves and late stage seeds. Further results indicated that seeds of *B. napus* with a high oil content show higher expression levels under drought stress conditions, suggesting that BnaVOCs not only respond to abiotic stress but may also affect lipid metabolism in drought stress. This present study provides a comprehensive overview of the *VOC* gene family and provides new insights into their biological function in *B. napus* evolution.

## Introduction

Vicinal oxygen chelate proteins (VOC) are members of an enzyme superfamily that could catalyse reactions with a common mechanistic attribute that is enabled by certain conserved active site residues. These residues perform the same functions in all members of the superfamily (Gerlt and Babbitt, 2001). The bidentate coordination to a divalent metal center through vicinal oxygen atoms is essential for activation or stabilization and is necessary for the functional mechanism of the VOC superfamily (He and Moran, 2011). In this superfamily, the coordination is mediated by a topological structure (βαβββ modules) that offers a ligand that shields a metal ion (Armstrong, 2000).

Based on the chemistry of the catalyzing enzyme, the VOC superfamily was divided into non-dioxygenase and dioxygenase groups. The non-dioxygenase group includes two families (isomerase and nucleophilic addition) that can catalyse non-redox active reactions. Because the metal center of dioxygenase has many function, such as activating dioxygen and organizing the co-substrates, dioxygenase also contain two families: extradiol dioxygenase and α-keto acid oxygenase (He and Moran, 2011). The first studied members of the VOC superfamily were glyoxalase I (GLYI), fosfomycin resistance protein (FosA), the related fosfomycin resistance protein X (FosX) and extradiol dioxygenases (EXDXs); EXDXs include 2,3-dihydroxybiphenyl1,2-dioxygenase (DHBD), catechol 2,3-dioxygenase (C23O), and homoprotocatechuate 2,3-dioxygenase (HPCD; Bernat et al., 1997). In 2000, methylmalonyl-CoA epimerase (MMCE) was added to the VOC superfamily based on its sequence and biochemical analysis (Armstrong, 2000). Then, the structures of 4-hydroxyphenylpyruvate dioxygenase (HPPD) and hydroxymandelate synthase (HMS) were also identified, and it was recognized that they should be considered members of the VOC superfamily (McCarthy et al., 2001; Brownlee et al., 2008).

GLYI is an important metalloenzyme that participates in the glyoxalase system, which has been reported to be a major pathway for detoxification of methylglyoxal (MG) in living organisms (Thornalley, 1990). MG is formed as a cytotoxic α-ketoaldehyde by-product of carbohydrate and lipid metabolism. MG reacts with nucleic acid molecules and protein to form adducts and can be harmful to organisms. GLYI can use one molecule of glutathione (GSH) to convert MG to S-Dlactoylglutathione (Singla-Pareek et al., 2003). The over-expression of GLYI cloned from *Brassica juncea* could lead to improved abiotic stress tolerance in transgenic tobacco (Reddy and Sopory, 1999). *Gly I* is a gene that is induced by drought and cold stress in *Arabidopsis* (Seki et al., 2001). Nineteen and twenty-two GLYIs have been identified in rice and *Arabidopsis thaliana*, respectively, and further research studies have indicated that these GLYIs were highly expressed under abiotic stress (Mustafiz et al., 2011). HPPD is another member of the VOC superfamily, and the constitutive over-expression of the barley *HPPD* gene could enhance the vitamin E content in transgenic tobacco seeds (Falk et al., 2003). The expression of the barley *HPPD* gene during senescence is most likely related to oxidative stress (Falk et al., 2002). In addition, a member of the VOC superfamily has been reported to be up-regulated during desiccation in the leaves, roots, and seeds of the resurrection plant *Xerophyta humilis* (Mulako et al., 2008). In addition, the desiccation induced-1VOC (dsi-1^VOC^) protein was also observed in *Brassica napus* with a high oil content, suggesting that it might protect the embryonic developmental process from harm under drought conditions (Gan et al., 2013).

*B. napus* (AACC, 2*n* = 38) is an allopolyploid species with a triplicated genome structure and many duplicated genes, which originated from *Brassica rapa* (AA, 2*n* = 20) and *Brassica oleracea* (CC, 2*n* = 18; Allender and King, 2010). *B. napus* is the third largest oil seed crop in the world. However, few studies have focused on the VOC superfamily in *B. napus*, except for the reported appearance of the VOC protein in high oil content *B. napus* lines (Gan et al., 2013), suggesting that the VOC protein may contribute to dehydration tolerance during the oil-accumulation period and help increase the oil content in *B. napus*. Because *B. napus* originated from hybridization, its genome contains many inversions or translocations and duplications (Chalhoub et al., 2014). Mesopolyploidization events were identified in Brassicaceae evolution, and increasing drought and transient glaciation events coincided with the Brassicaceae major evolutionary splits. Genomic differentiation events resulted in whole genome triplication (WGT), which formed *B. napus* (Cheng et al., 2014). Structural and functional divergence of duplicated genes within gene families were also found in other gene families, such as the mega-6 fatty acid desaturase (FAD2) gene family, the 12-oxo-phytodienoic acid reductases (OPRs) gene family, and the NBS-encoding gene family (Schlueter et al., 2007; Li et al., 2009; Yu et al., 2014). The characteristics of the duplicated genes suggest that evolution could have caused an adaptive structural diversification, and this process is pervasive and could have contributed to the biological novelty in plants (Li et al., 2009). Thus, many duplicated genes have similar gene sequences but different functional performances, and the large number of duplicated genes in polyploid plants complicates phylogenetic and evolutionary analyses. In addition, the structural and functional divergence of duplicated genes within a gene family could offer support for the gene expansion pattern in the species evolution (Liang et al., 2016). A systemic analysis of the VOC superfamily in *B. napus* has not been reported. In this study, the VOC superfamily in *B. napus* was identified, and its structure, evolution and synteny relationship with *BnaGLYIs* and *BnaHPPDs* were analyzed. In addition, the expression pattern of VOC superfamily members in different tissues of *B. napus* were also analyzed. This research study provides a foundation for future studies regarding the VOC superfamily in *B. napus*.

## Materials and methods

### Identification of VOC family genes in *B. napus* and other species

The *VOC* genes were identified in *B. napus* based on their homology with the 22 GLYI protein (Mustafiz et al., 2011) and 1 HPPD sequences from *Arabidopsis* using the BLAT search program in the CNS-Genoscope database (http://www.genoscope.cns.fr/brassicanapus/; Chalhoub et al., 2014). Redundant sequences were removed manually. The *VOC* genes in *B. rapa, B. oleracea, Brassica nigra* were obtained from the Brad database (http://brassicadb.org/brad/; Wang et al., 2011). All *BnaVOC* gene candidates were analyzed using the Hidden Markov Model (HMM), which is a statistical Markov model in which the modeled system is assumed to be a Markov process with unobserved (hidden) states; for this analysis, the following three databases were used for confirmation: the Pfam database (http://pfam.sanger.ac.uk/search; Finn et al., 2010), SMART database (http://smart.embl-heidelberg.de/; Letunic et al., 2004), and NCBI Conserved Domain Search database (http://www.ncbi.nlm.nih.gov/Structure/cdd/wrpsb.cgi; Marchler-Bauer et al., 2015).

A univocal name consisting of two italic letters denoting the source organism, the family name, and subfamily numeral of each gene was assigned to each *VOC* gene (e.g., *BnaGLYI1*; Ostergaard and King, 2008). The number of amino acids, CDS lengths, and chromosome locations of the *BnaVOC* genes were obtained from the *B. napus* database.

The physicochemical parameters, including the molecular weight (kDa) and pI, of each BnaVOC protein were calculated using the compute pI/Mw tool in ExPASy (http://www.expasy.org/tools/). GRAVY (grand average of hydropathy) values were calculated using the PROTPARAM tool (http://web.expasy.org/protparam/; Gasteiger et al., 2003). The subcellular location predictions were conducted using the TargetP1.1 (http://www.cbs.dtu.dk/services/TargetP/) server (Emanuelsson et al., 2007) and Protein Prowler Subcellular Localization Predictor version 1.2 (http://bioinf.scmb.uq.edu.au:8080/; Bodén and Hawkins, 2005).

### Multiple alignment and phylogenetic analysis of the VOC family genes

Multiple sequence alignment of all predicted BnaVOC, BrVOC, BoVOC protein sequences was performed using ClustalW software. An unrooted phylogenetic tree of these full-length VOC protein sequences was constructed using MEGA 6 with the Neighbor Joining (NJ) method, and a bootstrap analysis was conducted using 1,000 replicates (Higgins and Sharp, 1988; Tamura et al., 2013).

### Gene structure analysis of the *BnaVOC* family genes

The exon-intron structures of the *BnaVOC* family genes were determined based on the alignments of their coding sequences with the corresponding genomic sequences, and a diagram was created using GSDS (Gene structure display server: http://gsds.cbi.pku.edu.cn/; Hu et al., 2015). MEME (Multiple Expectation Maximization for Motif Elicitation; http://alternate.meme-suite.org/) was used to identify the conserved motif structures encoded by the BnaVOC family genes (Bailey et al., 2009). A secondary structure analysis was carried out using the following two different tools: PBIL GOR4 (Jones, 1999) and PSIPRED (Buchan et al., 2013). The Tertiary structures of the BnaVOCs were predicted using Phyre2 (Kelley and Sternberg, 2009), and they were analyzed using VAST.

### Chromosomal location and gene duplication of the *BnaVOC* family genes

The chromosomal locations of the *BnaVOC* genes were determined based on the positional information obtained from the *B. napus* database. Tandemly duplicated VOC genes were defined as adjacent to homologous *VOC* genes on *B. napus* chromosomes or within a sequence distance of 50 kb (Yu et al., 2014). The synteny relationships between the *BnaVOC*s and *AtVOC*s, *BrVOC*s, and *BoVOC*s were evaluated using the search syntenic genes tool in BRAD (http://brassicadb.org/brad/; Wang et al., 2011) and the synteny tools in the *B. napus* Genome Browser (Chalhoub et al., 2014).

### Calculation of the Ka/Ks values in the *BnaVOC* family genes

The *VOC* gene sequences of each paralogous pair were first aligned using ClustalW. Files containing the multiple sequence alignments of the *VOC* gene sequences were then converted to a PHYLIP alignment using MEGA. Then, the converted sequence alignments were imported into the YN00 program in PAML to calculate the non-synonymous and synonymous substitution rates (Yang, 2007).

### Plant materials, drought treatment, RNA extraction, and qRT-PCR analysis

The late grown plants (40 days after pollination) from the high oil content (55.19%) and low oil content (36.49%) *B. napus* lines were moved into a green chamber at 25°C with a 16-h light/8-h dark photoperiod. After 10 days of adaption, they were treated. For the drought stress treatment, water was withheld from these plants for 30 days. After 10, 20, or 30 days of treatment, RNA was extracted from the siliques. An RNAprep Pure Plant Kit (Tiangen) was used to isolate the total RNA from each frozen sample, and first-strand cDNA was synthesized from the RNA using a PrimeScriptTM RT Master Mix Kit (TaKaRa) according to the manufacturer's instructions.

Gene-specific primers were designed using Primer5.0 (Table S3). Each reaction was carried out in triplicate with a reaction volume of 20 μl containing 1.6 μl of gene-specific primers (1.0 μM), 1.0 μl of cDNA, 10 μl of SYBR green (TaKaRa), and 7.4 μl of sterile distilled water. The PCR conditions were as follows: Stage 1: 95°C for 3 min; stage 2: 40 cycles of 15 s at 95°C and 45 s at 60°C; and stage 3: 95°C for 15 s, 60°C for 1 min, 95°C for 15 s. At stage 3, a melting curve was generated to estimate the specificity of the reactions. A housekeeping gene (*actin*) that is constitutively expressed in *B. napus* was used as a reference for the normalization and analyzed using an ABI3100 DNA sequencer (Applied Biosystems; Quantitation-Comparative: ΔΔCT); three biological replicates were performed for the qRT-PCR analysis (Kagale et al., 2007).

## Results

### Genome-wide identification of the VOC superfamily genes in the *B. napus* genome

In total, 38 genes in the *B. napus* genome were identified as VOC superfamily genes using the CNS-Genoscope database based on their homology with *GLYI* genes and *HPPD* genes from *Arabidopsis* (Table 1). The *GLYI* and *HPPD* families were observed, while the other seven subfamilies were not found in *B. napus*, and no homologous genes were reported in *Arabidopsis* (*FosA, FosX, DHBD, C23O, HPCD, MMCE*, and *HMS*). The GLYI family and HPPD family in *B. napus* contains 34 members and 4 members, and the members were named *BnaGLYI1*-*BnaGLYI34* and *BnaHPPD1*-*BnaHPPD4*, respectively. The physicochemical parameters of each *VOC* gene were calculated using ExPASy. Except for *BnaGLYI11* and *BnaGLYI12*, the VOC (BnaVOC) proteins in *B. napus* had a molecular mass <50 KDa. In total, 31 of the BnaVOC proteins had relatively low isoelectric points (pI < 7). Nearly all the BnaVOCs had a GRAVY value < 0, indicating that a large proportion of the BnaVOC proteins are hydrophilic. Low hydrophobicity is a feature observed in other drought stress induced proteins (Beck et al., 2007), suggesting that the BnaVOC proteins may play a role under drought stress conditions. PProwler and TargetP were used to predict the subcellular location of the 38 BnaVOC proteins; the subcellular locations of the different BnaVOC proteins were diverse, and some proteins are predicted to be located in secretory pathways and the nucleus (Table S1).

**Table 1:** *****VOC*** genes in ***B. napus*** genome and their sequence characteristics and subcellular location prediction**.

**Name**	**Gene ID**	**Family**	**Chr**.	**Gene position**		**Gene length (bp)**	**protein length (aa)**	**Mol.Wt. (KD)**	**pI**	**GRAVY**	**Intron number**	**Subcellular location PProwler TargetP**	
				**Start**	**End**								
BnaGLYI1	BnaA09g49270D	Glyoxalase	A9	32,814,199	32,815,336	1,138	137	15.3942	5.84	−0.4635036	2	Other	S
BnaGLYI2	BnaA06g04170D	Glyoxalase	A6	2,547,123	2,548,306	1,184	171	19.2747	7.77	−0.4046783	3	Other	S
BnaGLYI3	BnaC05g05340D	Glyoxalase	A5	2,609,787	2,610,529	743	138	15.5964	5.94	−0.4688405	2	Other	S
BnaGLYI4	BnaCnng38880D	Glyoxalase	Cn-R	37,521,585	37,522,696	1,112	137	15.5023	6.2	−0.5124087	2	Other	S
BnaGLYI5	BnaC05g05770D	Glyoxalase	C5	2,848,240	2,850,316	2,077	235	26.4389	8.74	−0.4885106	7	C	C
BnaGLYI6	BnaA06g04580D	Glyoxalase	A6	2,708,014	2,710,119	2,106	237	26.6351	8.33	−0.4206751	7	C	C
BnaGLYI7	BnaC03g51010D	Glyoxalase	C3	35,479,070	35,480,133	1,064	150	16.9921	5.25	−0.4893333	4	Other	O
BnaGLYI8	BnaCnng47290D	Glyoxalase	Cn-R	46,764,251	46,765,432	1,182	143	16.1803	5.39	−0.4349650	5	Other	
BnaGLYI9	BnaC08g15100D	Glyoxalase	C8	19,658,835	19,661,229	2,395	283	31.8142	5.26	−0.3279151	7	Other	O
BnaGLYI10	BnaA08g25110D	Glyoxalase	A8	17,357,805	17,359,871	2,067	283	31.8724	5.26	−0.3215547	7	Other	O
BnaGLYI11	BnaC05g08770D	Glyoxalase	C5	4,659,651	4,665,014	5,364	724	80.6725	7.49	−0.2138121	19	M	M
BnaGLYI12	BnaA06g07360D	Glyoxalase	A6	3,915,659	3,923,909	8,251	1215	134.9846	8.86	−0.2618930	26	M	M
BnaGLYI13	BnaC05g11680D	Glyoxalase	C5	6,800,739	6,802,181	1,443	174	19.7337	5.68	−0.1994252	2	Other	O
BnaGLYI14	BnaA08g23870D	Glyoxalase	A8	1,684,3513	16,844,540	1,028	174	19.8469	5.88	−0.2264367	2	Other	O
BnaGLYI15	BnaA06g10060D	Glyoxalase	A6	5,342,612	5,343,786	1,175	174	19.7787	5.68	−0.2086206	2	Other	O
BnaGLYI16	BnaC08g16660D	Glyoxalase	C8	20,576,966	20,578,018	1,053	174	19.8218	5.89	−0.2862068	2	Other	O
BnaGLYI17	BnaA09g56790D	Glyoxalase	A9-R	3,889,968	3,891,223	1,256	167	18.9438	5.86	−0.2449101	2		M
BnaGLYI18	BnaC08g38920D	Glyoxalase	C8	34,930,459	34,931,660	1,202	173	19.5686	5.86	−0.2150289	2		
BnaGLYI19	BnaA02g19970D	Glyoxalase	A2	12,337,272	12,338,438	1,167	167	18.8186	5.81	−0.2095808	2	Other	O
BnaGLYI20	BnaC02g46640D	Glyoxalase	C2-R	2,394,713	2,395,834	1,122	167	18.8046	5.66	−0.2071856	2	Other	O
BnaGLYI21	BnaC02g23290D	Glyoxalase	C2	20,281,012	20,282,133	1,122	167	18.7625	5.66	−0.2347305	2	Other	O
BnaGLYI22	BnaC09g12330D	Glyoxalase	C9	8,747,895	8,748,643	749	118	12.9659	6.4	0.09152542	2	M	M
BnaGLYI23	BnaA09g12000D	Glyoxalase	A9	6,301,614	6,302,406	793	118	12.9839	6.4	0.04745762	2	M	M
BnaGLYI24	BnaC09g11920D	Glyoxalase	C9	8,321,491	8,322,355	865	118	12.9839	6.4	0.02966101	2	M	M
BnaGLYI25	BnaC06g28360D	Glyoxalase	C6	29,576,211	29,578,606	2,396	345	38.3689	6.19	−0.2481159	8	C	C
BnaGLYI26	BnaA07g26290D	Glyoxalase	A7	19,358,162	19,361,477	3,316	341	37.8944	6.48	−0.2533724	8	C	C
BnaGLYI27	BnaC04g15790D	Glyoxalase	C4	13,701,067	13,701,624	558	34	3.8642	6.7	−1.6470588	1	Other	O
BnaGLYI28	BnaA07g13890D	Glyoxalase	A7	12,263,440	12,263,994	555	184	2.0887	4.78	−0.6913043	0	Other	O
BnaGLYI29	BnaA05g35240D	Glyoxalase	A5-R	670,044	672,049	2,006	138	15.2522	5.46	−0.2255474	3	Other	O
BnaGLYI30	BnaCnng59150D	Glyoxalase	Cn-R	58,862,580	58,863,862	1,283	137	15.2531	5.45	−0.2788321	3	Other	O
BnaGLYI31	BnaA09g11460D	Glyoxalase	A9	5,894,872	5,895,565	694	118	12.9378	6.4	0.07288135	2	M	M
BnaGLYI32	BnaA10g11070D	Glyoxalase	A10	9,346,288	9,347,612	1,325	195	21.959	6.71	−0.4492307	3		O
BnaGLYI33	BnaC03g13130D	Glyoxalase	C3	6,313,042	6,314,254	1,213	193	21.7128	7.77	−0.4398963	3		O
BnaGLYI34	BnaA03g10440D	Glyoxalase	A3	4,700,661	4,701,804	1,144	193	21.7859	8.43	−0.4880829	3		C
BnaHPPD1	BnaC05g04530D	HPPD	C5	2,231,485	2,233,489	2,005	445	48.8579	5.65	−0.2617977	1	Other	O
BnaHPPD2	BnaA09g49870D	HPPD	A9	33,093,200	33,095,241	2,042	440	48.0721	5.72	−0.2372727	1	Other	O
BnaHPPD3	BnaC08g44820D	HPPD	C8	37,849,659	37,851,160	1,502	440	48.1622	5.46	−0.2440909	1	Other	O
BnaHPPD4	BnaA10g04310D	HPPD	A10	2,268,795	2,272,272	3,478	587	48.973	5.45	−0.2878651	11	Other	O

### Sequence alignment and phylogenetic analysis of the VOC genes in *B. napus, B.rapa, B. oleracea, B. nigra*, and *A. thaliana*

**Figure 1:**
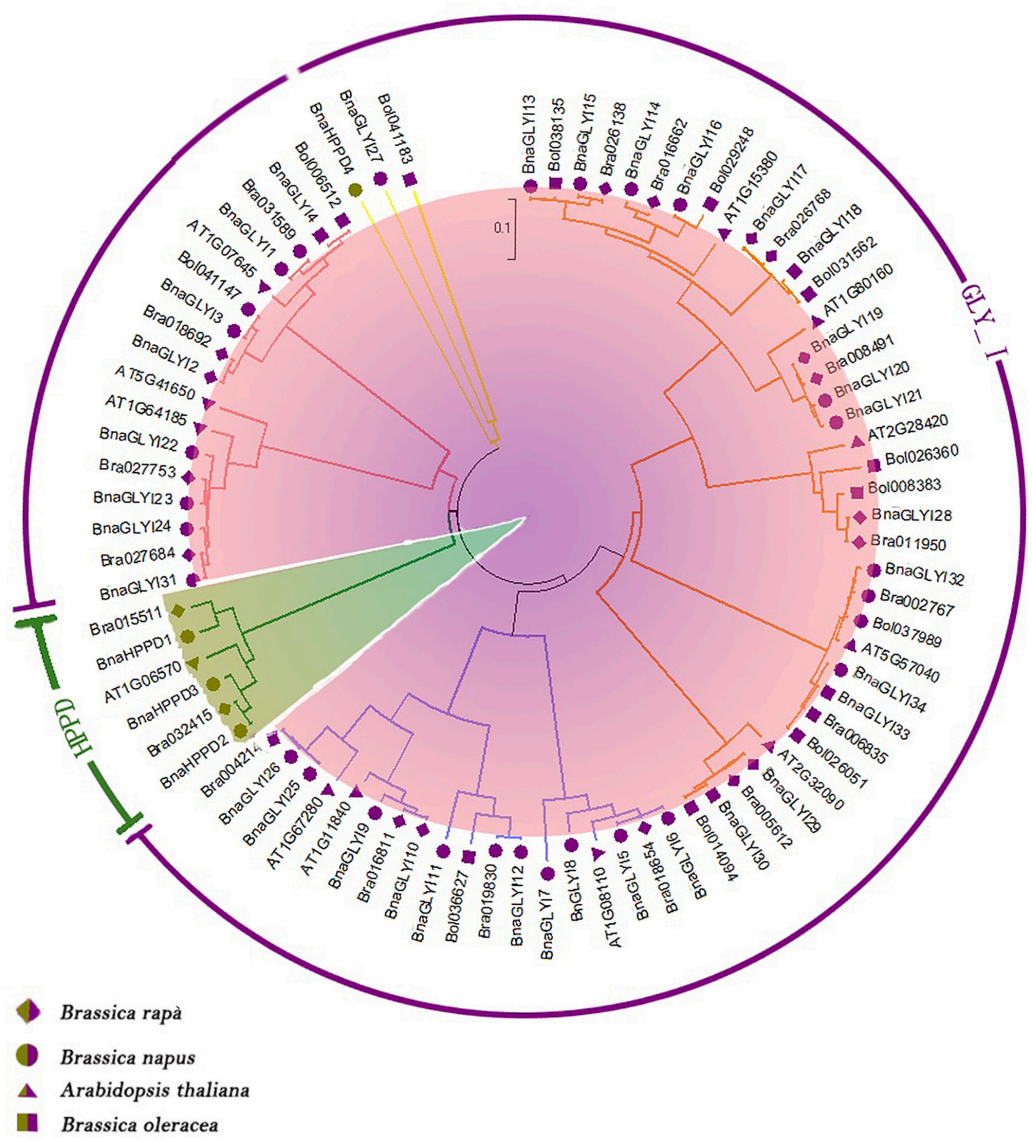
**Phylogenetic analysis of the ***B. napus, B. rapa, B. oleracea***, and ***A. thaliana VOC*** genes**. *VOC* gene families are distinguished by different colors. The unrooted tree was generated by using ClustalW in MEGA with the full-length amino acid sequences of the VOC proteins.

To determine the similarity and homology of the *BnaVOC* genes in *B. napus* and other brassica species, sequence alignments and a phylogenetic analysis were performed. The *GLYI* genes and *HPPD* genes in *B. rapa, B. oleracea*, and *A. thaliana* were used to construct an unrooted phylogenetic tree (Figure 1). Eighteen and ten homologous genes were identified in *B. rapa* and *B. oleracea*, respectively (Figure 1, Table S4). All *VOC* gene sequences clustered into five main clades, and almost half of the *GLYI* genes were clustered in one branch. Furthermore, the *VOC* genes in another diploid *Brassica* species, *Brassica nigra* (*B. nigra*), were also analyzed. Fourteen homologous *VOC* genes were detected in *B. nigra* (Table S4). Interestingly, *BnaGLYI27, Bol041183*, and *BnaHPPD4* were in the same clade, but this clade has a long branch length; on the one hand, this clade might result from the characteristics of their sequences because they contain consensus sequences; on the other hand, this result suggested that the divergence has not occurred in recent years. Almost all the *B. napus VOC* genes appeared as pair-wises with the *VOC* genes in *B. oleracea* or *B. rapa* in the phylogenetic relationship, and they contained long branch lengths, indicating that they have been evolved for a long time. For the phylogenetic analysis of the *Brassica* species, the *VOC* genes in *B. nigra* were usually in a different clade from the pair-wise genes, except for BniB014868, BniB034245, and BniB003628 (Figure S4). This result suggested that *B. nigra* had a farther relationship with *B. rapa, B. oleracea*, and *B. napus*, but *BnaHPPD1, BnaGLYI7*, and Bol026360 may be more closely related to *B. nigra*. While the *AtVOC* genes were in the same clade, they were on opposite sides, which is consistent with the evolutionary history of these *Brassica* plants. Altogether, these results indicated that in the *VOC* gene family, *BnaVOC*s, *BoVOC*s, and *BrVOC*s have a high homology. Some genes (*BnaGLYI 27* and *BnaHPPD4*) were in the same clade, but they had different conserved domains, suggesting that the other parts of these genes have evolved a closer phylogenetic relationship.

### Chromosomal location and expansion pattern analysis of the *VOC* genes in *B. napus*

**Figure 2:**
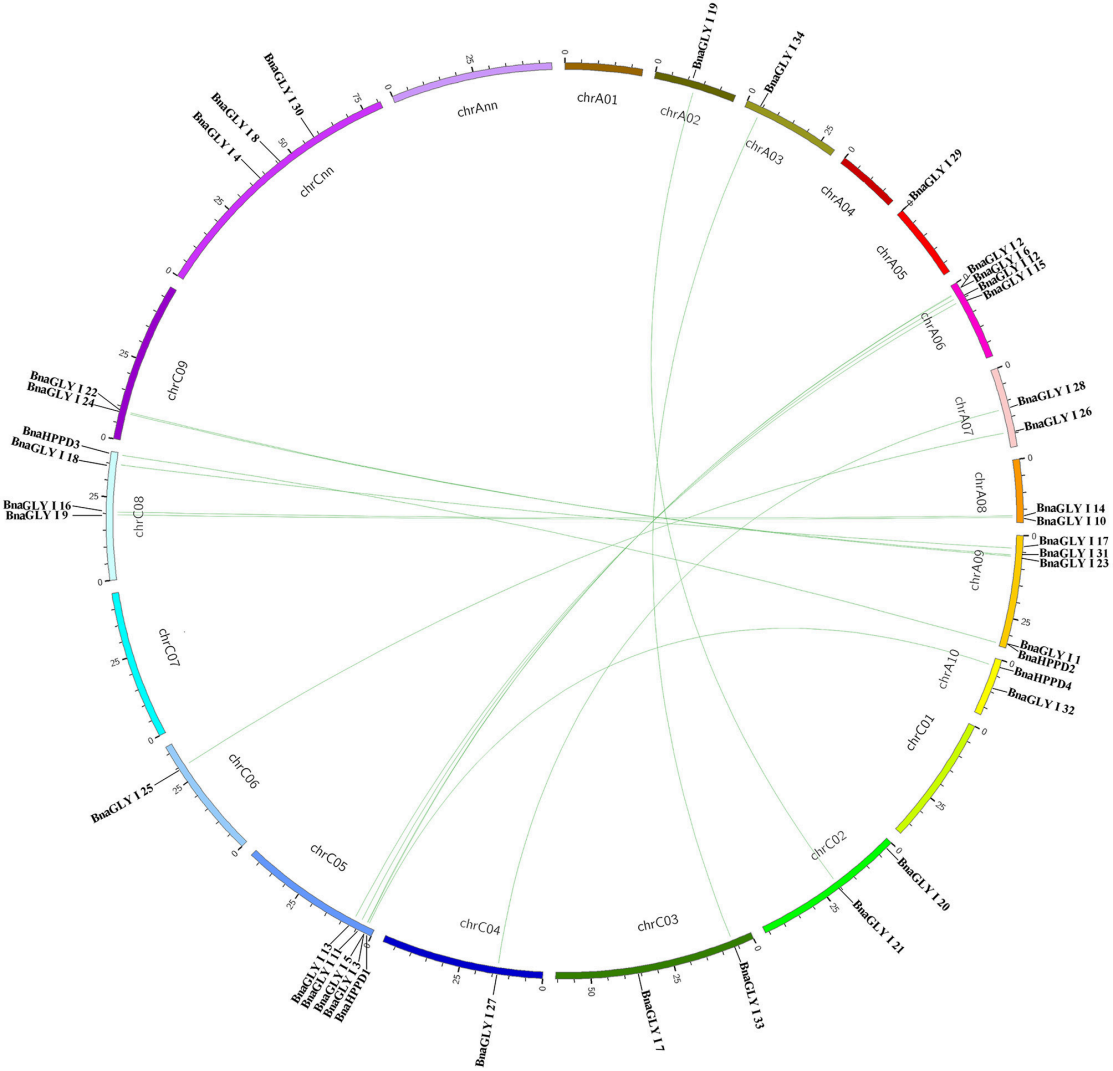
**Distribution of ***BnVOC*** gene family members on ***B. napus*** chromosomes**. The *BnVOC* genes that chromosomal information was available in the database were mapped to the *B. napus* chromosomes and synteny relationship were lined.

**Figure 3:**
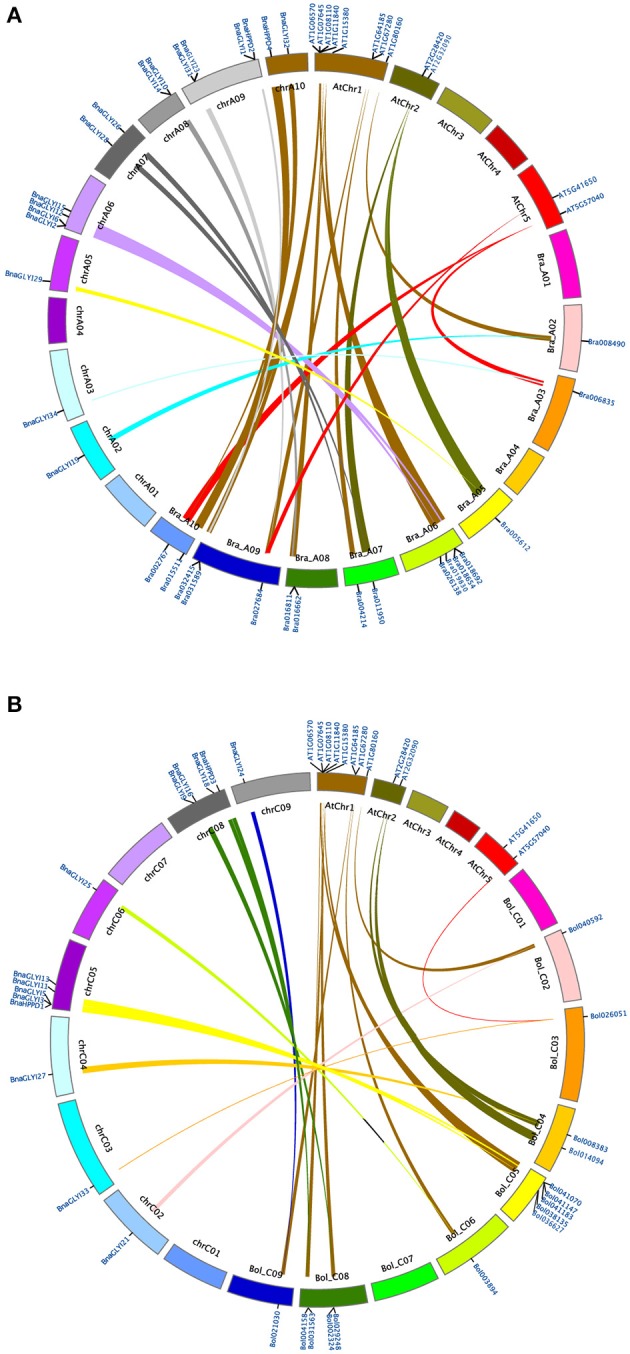
**Synteny analysis map of ***VOC*** gene in ***B. napus***, ***B. rapa, B. oleracea***, and ***A. thaliana*** chromosomes. (A)** Genes located on *B. napus* A genome are syntenic with genes of *B. rapa* and *A. thaliana*. **(B)** Genes located on *B. napus* C genome are syntenic with genes of *B. oleracea* and *A. thaliana*. The different degrees of thickness of lines indicate their synteny relationship (thick, strong; thin, weak).

The chromosomal location of the *BnaVOC* genes was analyzed, and the positions and chromosome locations of 35 *BnVOC* genes were clearly identified on the 14 chromosomes in *B. napus* (Table 1, Figure 2). The number of *BnaVOC* genes varies among the different chromosomes, and no *BnaVOC* genes were observed on chromosome A1, A4, C1, and C7. Chromosome C5 contains the greatest number of *BnaVOC* genes, and the five *BnaVOC* genes that are located on chromosome C5 appear as a gene cluster. The final chromosomal location may result from *VOC* gene duplication in the long evolutionary history. Compared with *A. thaliana, Brassica* species experienced an extra WGT event, and the WGT event contributed to a gene-level evolution and drove the diversification of the *Brassica* plants (Cheng et al., 2014). In addition, the homology synteny and chromosomal gene location analysis revealed that the *BnaVOC* genes are closely phylogenetically related to other *VOC* genes in *Brassicaceae* species (*B. oleracea, B. rapa*, and *A. thaliana*; Figures 1, 3). No tandemly duplicated genes were identified in the *BnaVOC* gene family, and 31 *BnaVOC* genes are associated with segmental duplications (Figure 2). Two loci (At1g11840 and At1g06570) had four copies that are involved in segmental duplications (Table 2). One loci, At1g15380, contains eight copies that are associated with segmental duplications (Table 2); these results indicate that segmental duplication played an important role in the gene evolution of this loci. Interestingly, the *BnaVOC* genes that clustered on chromosome A6 were closely linked with the genes located on chromosome C5, suggesting that the fragment in this region experienced segmental duplication. In addition, all the *VOC* genes in the *Brassica* species have a syntenic relationship with the chromosome of translocation Proto-Calepineae Karyotype (tPCK), which is an ancestral genome of the *Brassica* species. AT1G07645, AT1G08110, AT1G11840, AT1G15380, AT1G06570 and the related *Brassica* genes were identified from tPCK1, and genes from AT1G80160 and AT1G67280 were linked with tPCK6, AT2G28420, and AT5G57040, which had synteny relationship with tPCK3 and tPCK5. The synteny relationship between tPCK and the *Brassica* species indicated that the *VOC* genes evolved from different ancestral genomes and expanded to diverse chromosomes through segmental duplications.

**Table 2:** **Non-synonymous (Ka) and synonymous (Ks) nucleotide substitution rates for ***Arabidopsis thaliana*** and ***B. napus*** VOC gene coding loci**.

***A. thaliana* ID**	***B. napus* gene**	***B. napus* ID**	**VOC family**	**Ka**	**Ks**	**Ka/Ks**
**ONE COPY LOCI**						
AT5G41650	BnaGLYI31	BnaA09g11460D	Glyoxalase	0.1318	0.0949	0.7202
**TWO-COPY LOCI**						
AT1G67280	BnaGLYI25	BnaC06g28360D	Glyoxalase	0.1413	0.0598	0.4232
	BnaGLYI26	BnaA07g26290D	Glyoxalase	0.1499	0.0605	0.4039
AT2G28420	BnaGLYI27	BnaC04g15790D	Glyoxalase	0.3604	0.2398	0.6654
	BnaGLYI28	BnaA07g13890D	Glyoxalase	0.3669	0.2534	0.6907
AT2G32090	BnaGLYI29	BnaA05g35240D	Glyoxalase	0.0607	0.0328	0.5402
	BnaGLYI30	BnaCnng59150D	Glyoxalase	0.071	0.0327	0.4612
**THREE-COPY LOCI**						
AT1G64185	BnaGLYI22	BnaC09g12330D	Glyoxalase	0.0669	0.0306	0.4579
	BnaGLYI23	BnaA09g12000D	Glyoxalase	0.0829	0.0346	0.417
	BnaGLYI24	BnaC09g11920D	Glyoxalase	0.0745	0.0386	0.5178
AT5G57040	BnaGLYI32	BnaA10g11070D	Glyoxalase	0.1294	0.0564	0.4361
	BnaGLYI33	BnaC03g13130D	Glyoxalase	0.1193	0.0599	0.5023
	BnaGLYI34	BnaA03g10440D	Glyoxalase	0.1183	0.0649	0.5486
**FOUR-COPY LOCI**						
AT1G07645	BnaGLYI1	BnaA09g49270D	Glyoxalase	0.0534	0.0274	0.5133
	BnaGLYI2	BnaA06g04170D	Glyoxalase	0.0963	0.0494	0.5124
	BnaGLYI3	BnaC05g05340D	Glyoxalase	0.1078	0.0493	0.4571
	BnaGLYI4	BnaCnng38880D	Glyoxalase	0.0606	0.0291	0.4807
AT1G08110	BnaGLYI5	BnaC05g05770D	Glyoxalase	0.0491	0.0206	0.4196
	BnaGLYI6	BnaA06g04580D	Glyoxalase	0.0546	0.0206	0.3771
	BnaGLYI7	BnaC03g51010D	Glyoxalase	0.4269	0.2508	0.5874
	BnaGLYI8	BnaCnng47290D	Glyoxalase	0.1701	0.0679	0.3992
AT1G11840	BnaGLYI9	BnaC08g15100D	Glyoxalase	0.0986	0.0341	0.3459
	BnaGLYI10	BnaA08g25110D	Glyoxalase	0.0357	0.0056	0.1561
	BnaGLYI11	BnaC05g08770D	Glyoxalase	0.4815	0.1503	0.312
	BnaGLYI12	BnaA06g07360D	Glyoxalase	0.2625	0.0884	0.3368
AT1G06570	BnaHPPD1	BnaC05g04530D	HPPD	0.0607	0.0553	0.9118
	BnaHPPD2	BnaA09g49870D	HPPD	0.048	0.0461	0.9598
	BnaHPPD3	BnaC08g44820D	HPPD	0.0531	0.0507	0.9549
	BnaHPPD4	BnaA10g04310D	HPPD	0.6695	1.767	2.6391
**NINE-COPY LOCI**						
AT1G15380	BnaGLYI13	BnaC05g11680D	Glyoxalase	0.1089	0.0543	0.4983
	BnaGLYI14	BnaA08g23870D	Glyoxalase	0.1361	0.0583	0.4285
	BnaGLYI15	BnaA06g10060D	Glyoxalase	0.1152	0.0598	0.5195
	BnaGLYI16	BnaC08g16660D	Glyoxalase	0.1445	0.0613	0.424
	BnaGLYI17	BnaA09g56790D	Glyoxalase	0.1976	0.0887	0.449
	BnaGLYI18	BnaC08g38920D	Glyoxalase	0.2202	0.0917	0.4162
	BnaGLYI19	BnaA02g19970D	Glyoxalase	0.0747	0.0864	1.1556
	BnaGLYI20	BnaC02g46640D	Glyoxalase	0.0764	0.0922	1.2066
	BnaGLYI21	BnaC02g23290D	Glyoxalase	0.0819	0.0952	1.1624

**Figure 4:**
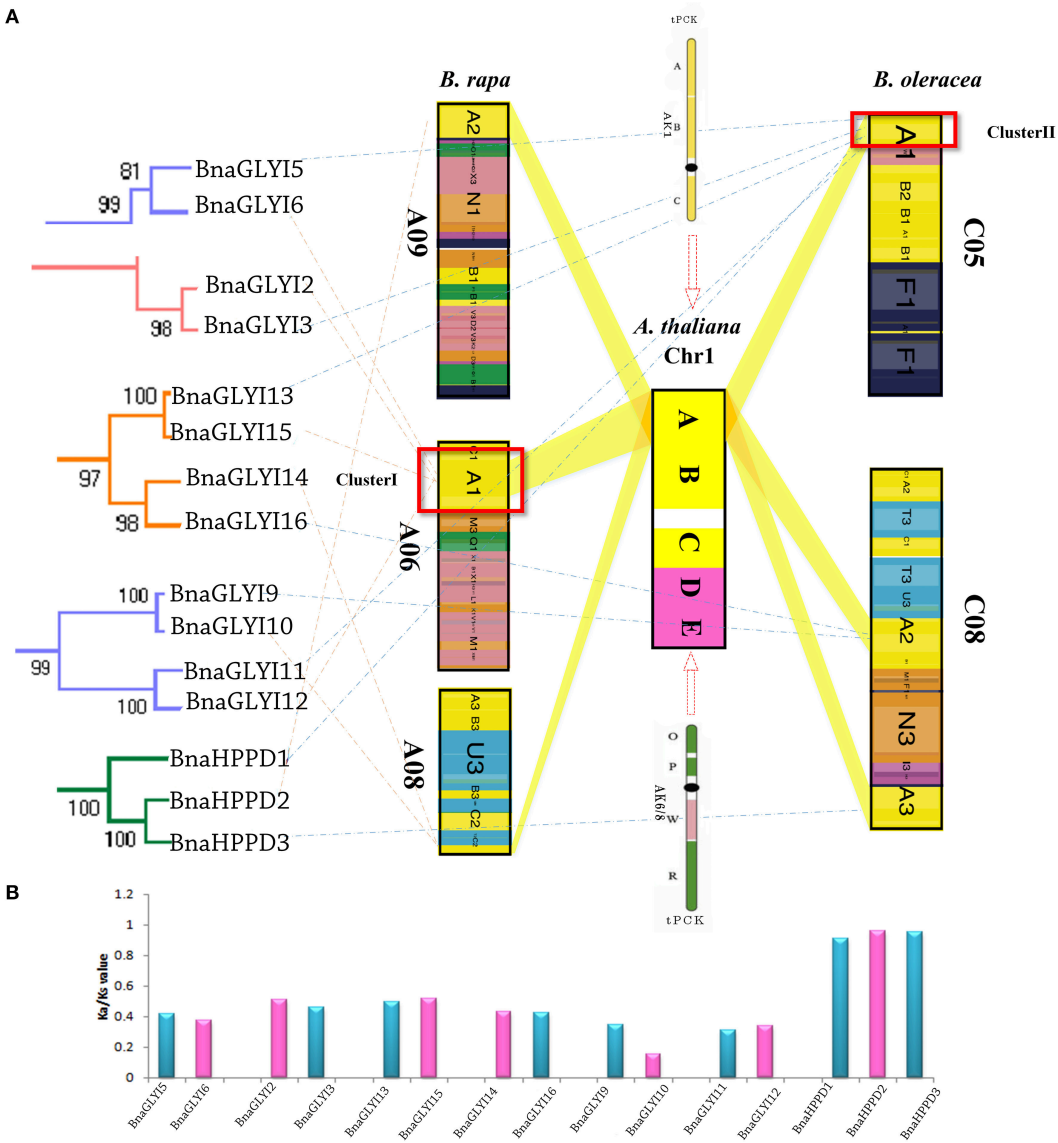
**Evolutionary pattern analysis of pair-wised ***BnaVOC*** genes. (A)** Phylogenetic relationships and evolutionary progress of the *VOC* genes in *Brassica* evolution. **(B)** Ka/Ks value comparison of the pair-wised *BnaVOC* genes.

By comparing the distributions of the genes around the *VOC* genes in the genomes of *A. thaliana, B. rapa, B. oleracea*, and *B. napus*, the synteny of the GLY I and HPPD families was revealed to be preserved, and some genes were either duplicated or lost. Furthermore, the synteny maps of the homologous genes in *A. thaliana, B. rapa* and the *BnaVOC* genes in *B. napus* genome A and the homologous genes in *A. thaliana, B. oleracea* and the *BnaVOC* genes in *B. napus* genome C were analyzed (Figure 3); it was revealed that most of homologous *VOC* genes were clustered on top of *Arabidopsis* chromosome 1; these *AtVOC* genes had synteny relationships with the *BrVOC* and *BoVOC* genes; and the *AtVOC* genes were duplicated and distributed to the *BrVOC*s or *BoVOC*s located on different *B. rapa* or *B. oleracea* chromosomes. In addition, nearly all of these homologous *BrVOC*s or *BoVOC*s genes maintained a synteny relationship with *BnaVOC*s (Figure 3). Interestingly, most of the *BnaVOC*s appear as pair-wises for more than 90% of the *AtVOC*s that have synteny relationships with two or more *BrVOC*s or *BoVOC*s. Further analysis revealed that this phenomenon was an evolutionary result of genome rearrangement after WGT (Figure 4). Most phylogenetic pair-wise *BnaVOC*s share an ancestral gene on *A. thaliana* chromosome 1, and these *AtVOC* genes were syntenic linked with tPCK1 or tPCK6 (Figure 4). Abundant genome rearrangements caused triplicated ancestral genomic blocks in the A and C genomes of *B. rapa* and *B. oleracea* (Liu et al., 2014), and *B. napus* was formed by allopolyploidy; the *VOC* genes in *B. napus* were syntenically linked with the genes in *B. rapa* and *B. oleracea*. This pattern can be observed not only in the *VOC* gene clusters that are located on the A6 and C5 chromosomes but also in the *VOC* genes that are located on other chromosomes. These findings suggested that the *VOC* gene family expansion pattern is a consequence of mesopolyploidy in *Brassica* evolution. Then, the non-synonymous (Ka) and synonymous (Ks) value were used to explore the selective pressure on the duplicated *BnaVOC* genes. Most *VOC* genes in the A genome have a higher Ka/Ks ratio than the homologous genes in the C genome (Figure 4, Table 2), suggesting that different evolution pressures existed in the *Brassica* species divergence evolution. In general, the Ka/Ks ratio indicates the different evolution pressures; a Ka/Ks ratio that is >1 indicates positive selection, while a Ka/Ks ratio that is <1 indicates a functional constraint, and a Ka/Ks ratio equal to 1 indicates neutral selection (Nekrutenko et al., 2002). The results revealed that most of the *BnaVOC* genes have Ka/Ks ratios >0.1 and <1. However, *BnaGLYI19, BnaGLYI20*, and *BnaGLYI21* had Ka/Ks ratios that were >1, suggesting that these three genes experienced positive selection pressure in evolution. The genes in the GLYI families exhibit relatively lower Ka/Ks ratios, whereas the *HPPD* gene families have higher Ka/Ks ratios. These findings indicated that most *BnaVOC* genes might preferentially conserve function and structure under selective pressure. *BnaGLYI19, BnaGLYI20*, and *BnaGLYI21* show positive selection, suggesting that they might develop a relatively functional formation. These results indicated that WGD and segmental duplication play a role in the *VOC* gene family evolution, which is consistent with the *Brassica* evolutionary process.

### Structure analysis in the *BnaVOC* genes

**Figure 5:**
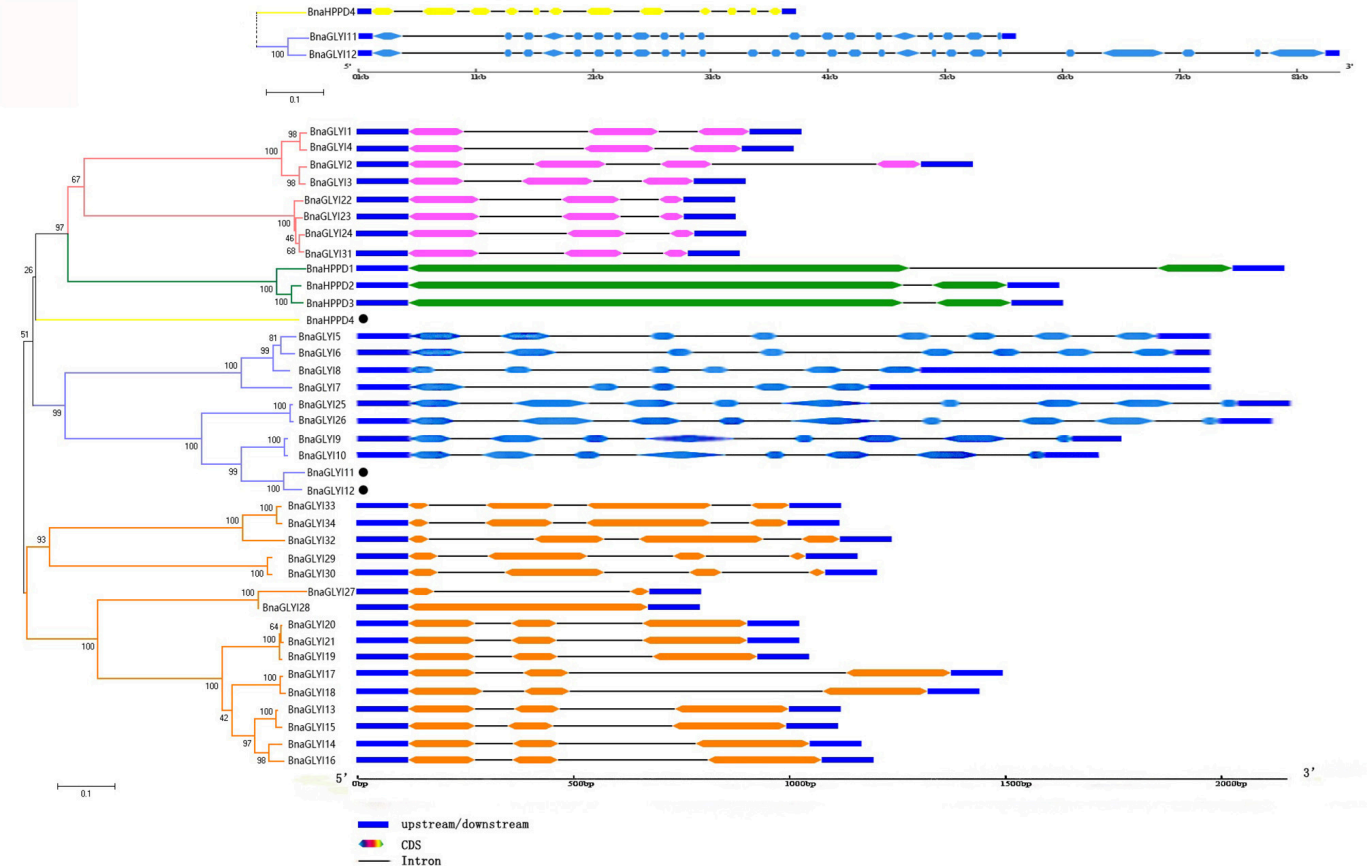
**Exon-intron organization of the ***BnaVOC*** genes**. Double-sided wedge boxes represent exons, and different colors indicate sub-clade families. Black lines represent introns, and untranslated regions (UTRs) are indicated by mazarine boxes. The lengths of the proteins and motifs can be estimated using the scale at the bottom.

The phylogenetic tree of the *B. napus VOC* genes shows four main clades, and *BnaHPPD4* is in a special clade. The lengths of most *BnaVOC* genes were shorter than 3 kb, except for *BnaHPPD4, BnaGLYI11* and *BnaGLYI12* (Figure 5). In the three main clades of the *BnaVOC* genes, the different clades contain different intron-exon structural features. Most of the *BnaVOC* genes have two or three introns, and more than three introns were observed in the *BnaVOC* genes that were longer than 1.5 kb. *BnaGLYI11* and *BnaGLYI12* were both longer than 5 kb, and it was revealed that these two genes have similar intron-exon structural characteristics with more than 10 introns; their intron-exon organization also reflects their close phylogenetic relationship. The same phenomenon is also observed in *BnaHPPD4*. Most *BnaVOC* genes were pair-wises, and their intron-exon structures are similar to that in the pair-wise *BnaVOC*s.

Because all the *BnaVOC*s genes have a low similarity, the genes in each clade were submitted to MEME for a motif structure analysis. Six motifs were identified to be conserved motifs, except for *BnaGLYI32, BnaGLYI33, BnaGLYI34*, and *BnaGLYI 27*. Motif 1 was present in each clade and encoded a conserved VOC family domain as indicated by the Pfam codes (Pfam000903 and Pfam 14696) and WebLogo (Figure S1). Most of the closely related genes in each phylogenetic branch exhibited similar motif compositions, suggesting the presence of functional similarities in the VOC family. *BnaGLYI11* and *BnaGLYI12*, which are longer than 2 kb, contain nine and eleven motifs, respectively. BnaGLYI9, BnaGLYI10, BnaGLYI25, and BnaGLYI26 are in the same clade and have shorter lengths, but they contain a higher number of motifs than the genes in the other clades (Figure S1). These results imply that the composition of the structural motifs varies among the different *VOC* genes but is similar within the same phylogenetic branch and that the motifs encoding the VOC domains are conserved.

**Figure 6:**
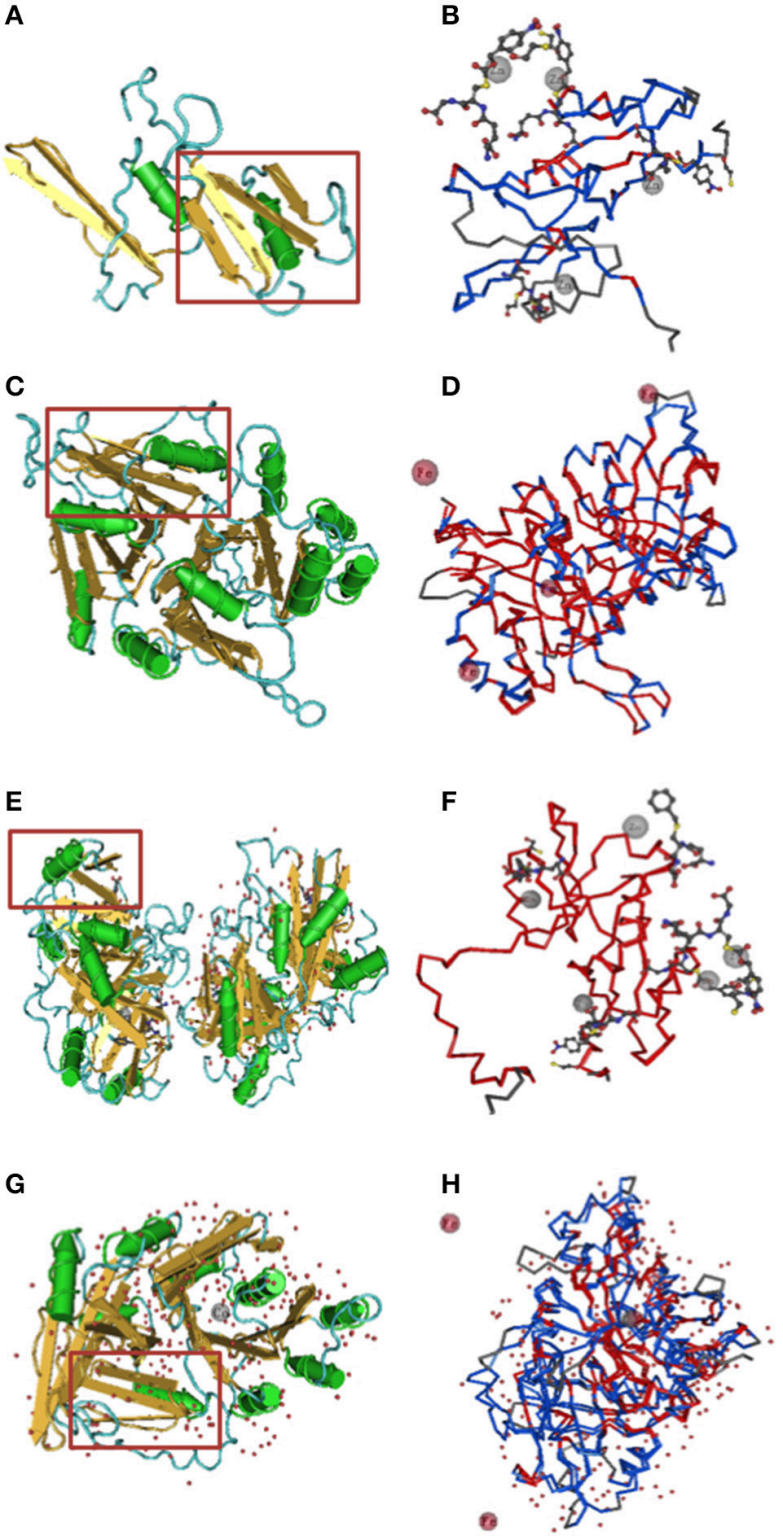
**Predictive 3D domain structure of BnaVOCs**. BnGLYI19 and BnHPPD2 were selected as examples to show. The models were generated from Phyre2 with 100% of confidence. Conserved domain analysis was highlighted using VAST. **(A)** Predictive 3D structure of BnGLYI19. **(B)** Conserved domains and chelated metal ion of BnGLYI19. **(C)** Predictive 3D structure of BnHPPD2. **(D)** Conserved domains and chelated metal ion of BnHPPD2. **(E)** 3D structure of human GLYI. **(F)** Conserved domains and chelated metal ion of human GLYI. **(G)** 3D structure of human HPPD. **(H)** Conserved domains and chelated metal ion of human HPPD. Red boxes indicate a typical structure of βαβββ.

The secondary structures and three-dimensional structures of the BnaVOCs were also analyzed. GOR4 and PSIPRED were used for the secondary structure prediction, and similar results were obtained (Figure S2). The BnaVOCs mainly contained α-helixes, extended strands and random coils. The α-helixes accounts for ~20%, and the strand structures account for ~30%. Based on the results from the GOR4 database, the VOC-special structure (βαβββ) was observed in these BnaVOCs sequences. Some BnaVOCs, such as BnaGLYI22 and BnaGLYI24, contain more at least two VOC-special structures (βαβββ). The three-dimensional structures of the BnaVOCs were modeled and predicted using the Phyre2 database. First, all protein sequences were analyzed by VAST, and then, their sequences were compared with the structures obtained from the Protein Data Bank. The predicted domains were separately presented in Cn3D macromolecular structure viewer, and the human glyoxalase I and HPPD structures were selected for the homology modeling because their structures were well-studied, clear, and have been used in modeling the VOC in *Arabidopsis* (Figure 6). The tertiary structures of the s βαβββ domains. Compared with the BnaGLY I proteins, the BnaHPPDs contain more than one βαβββ repeat. In addition, the divalent metal centers of the BnaGLY I proteins and BnaHPPD proteins were different, and the zinc ions in the BnaGLYI proteins were usually predicted to appear in the metal center; however, the BnaHPPD proteins were predicted to contain ferric ion in the center in the intermediate state through the vicinal oxygen atoms (Figure 6). These results suggest that the BnaVOC proteins have conserved structures but also show some differences, particularly in the divalent metal centers and the number of βαβββ folds. All these differences might contribute to their different functions in diverse environments.

### Expression profile analysis of the *BnaGLYI* and *BnaHPPD* genes in different tissues

To investigate the expression pattern of the VOC genes in *B. napus*, the root, stem, leaves, flower, early developmental stage seeds (24 weeks after seeding), and late stage developmental seeds (30 weeks after seeding) were used to perform a gene expression analysis using the qPCR technique. Because homologous *VOC* genes were mainly related to desiccation and drought stress, there are fewer reports regarding their function under other types of abiotic stress (Mulako et al., 2008; Mustafiz et al., 2014). *B. napus* were treated with drought stress to study the *BnaVOC* expression pattern under drought stress conditions; to determine whether the *BnaVOC*s expression is linked to lipid formation under drought condition, high oil content and low oil content *B. napus* were used, and the analysis was performed with qPCR analysis.

**Figure 7:**
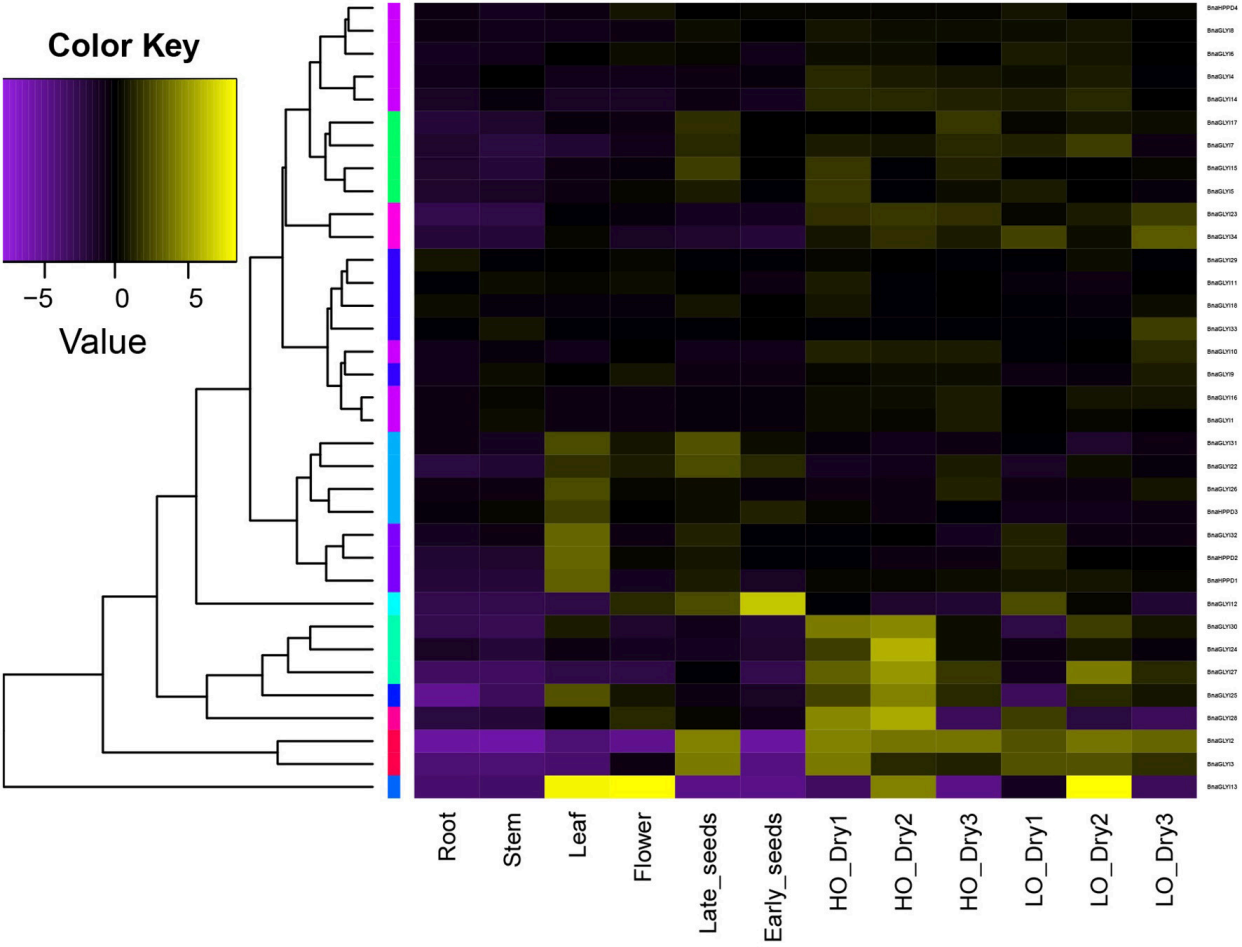
**Hierarchical clustering of the expression profiles of ***BnaVOC*** genes in different tissues and the seeds in drought stress**. The log-transformed values of the relative expression levels of *BnaVOC* genes were used for hierarchical cluster analysis (original data shown in Table S2). The color scale represents relative expression levels with increased transcript (yellow) or decreased transcript (purple). Late_stage seeds were drought stress treated (HO_Dry1, HO_Dry2, HO_Dry3 indicate high oil content *B. napus* lines were treated for 1, 2, 3 weeks, respectively; LO_Dry1, LO_Dry2, LO_Dry3 indicate low oil content *B. napus* lines were treated for 1, 2, 3 weeks, respectively).

Compared with the other organs, the leaf, and late developmental stage seeds show a higher expression level of *BnaVOC*s (Figure 7). The leaves are important organs for transpiration and are sensitive tissues under stress conditions (Xiong and Zhu, 2002); late developmental stage seeds frequently experience dehydration, and the high expression level of the *BnaVOC*s in these two tissues was consistent with reported proteins levels of the *VOC* gene family and their expression pattern in other plants (Mulako et al., 2008; Mustafiz et al., 2011). After the dry treatment, the expression level of most *BnaVOC* genes was higher in the high oil content *B. napus* seeds than that in the low oil content *B. napus* seeds. For example, *BnaGLYI28, BnaGLYI30, BnaGLYI24, BnaGLYI25, BnaGLYI10*, and *BnaHPPD3* (Figure 7, Table S2). Interestingly, during the dry treatment, different *BnaVOC* genes presented different expression patterns. Certain *BnaVOC* genes, such as *BnaGLYI12, BnaGLYI32*, and *BnaHPPD2*, showed a higher expression level during the early drought stress stage (HO_Dry1, LO_Dry1) and a lower expression level during the late drought stress stage and intermediate drought stress stage. Certain *BnaVOC* genes were highly expressed during the late drought stress stage (HO_Dry3, LO_Dry3), such as *BnaGLYI17, BnaGLYI26*, and *BnaGLYI33*. Many *BnaVOC* genes presented a higher expression during the intermediate drought stress stage (HO_Dry2, LO_Dry2), particularly *BnGLYI13*. Certain *BnaVOC* genes showed very different expression patterns between the high oil content seeds and low oil content seeds during the dry treatment, such as *BnaGLYI18, BnaGLYI10, BnaGLYI32*, and *BnaGLYI29*. These results suggest that these genes share a homologous structure, but they might function differently under drought stress conditions. In addition, because certain *BnaVOC* genes contain different expression features between the high and low oil content of *B. napus*, they might affect lipid metabolism and the final oil formation in *B. napus*. Most of the pair-wise genes showed similar expression patterns, but some phylogenetic gene pairs had different expression patterns (*BnaHPPD2*/*BnaHPPD3, BnaGLYI11*/*BnaGLYI12, BnaGLYI13*/*BnaGLYI15, BnaGLYI17*/*BnaGLYI18, BnaGLYI25*/*BnaGLYI26*, and *BnaGLYI29*/*BnaGLYI30*). This finding suggests that these pair-wise genes may have different functions in *B. napus*. These results indicate that even if the *BnaVOC* genes are diversely expressed in different tissues, their high expression level in the leaves shows their important roles in drought stress. When the different oil content *B. napus* lines were under the dry treatment, the *BnaVOC* genes showed different expression patterns between the two types of *B. napus*, and certain *BnaVOC* genes had a higher level of expression in the high oil content *B. napus*; this result suggests that the BnaVOC protein not only has a biological function in drought resistance but may also affect lipid metabolism and lead to the differences in the final oil content, which is consistent with the results of the proteomic analysis of the different oil content *B. napus* lines (Gan et al., 2013).

## Discussion

### Structural characteristics of the *BnaVOC* family

The VOC superfamily is a type of metalloenzyme superfamily; therefore, this superfamily contains structurally related proteins (Bergdoll et al., 1998). These VOC proteins can provide a metal coordination environment for the electrophilic participation of the metal ion in catalysis, and these proteins may participate in the evolution of protein folding (Armstrong, 2000; Gerlt and Babbitt, 2001). Some gene structures in the VOC superfamily have been reported in different species (Bernat et al., 1997; McCarthy et al., 2001; Thornalley, 2003); however, a genome-wide identification and annotation of the VOC genes have not been reported in *B. napus*. In this study, 38 genes were identified as VOC family genes in the *B. napus* genome. In general, gene families that are associated with stress resistance contain fewer introns (Liang et al., 2016), and our present results confirmed this conclusion to a certain extent in 27 of the *BnaVOC* genes that have no more than three introns. However, one clade of *BnaGLYI*s and *BnaHPPD4* had more than three introns, and this likely was due to their lengths, which were longer than 1,000 bp, and the number of βαβββ folds; many introns were conserved in evolution (Figure 1). The composition and motif numbers in each clade varied, but the conserved motif of Pfam 00903 or Pfam 14696 was observed in every member of the *BnaVOC* gene family. The differences in the motifs between BnaGLYIs and BnaHPPDs were likely due to their configurations in the functional form; for instance, glyoxalase I can exist as a dimer or a monomer (Saint-Jean et al., 1998). The three-dimensional structural analysis showed that Fe^2+^ and Zn^2+^ were in the metal center (Figure 6), and they were also found in the VOC family in other species (He and Moran, 2011; Mustafiz et al., 2011), supporting that the BnaVOCs can play important roles in catalytic function as metalloenzymes.

### Duplication patterns and synteny analysis of the *BnaVOC* super family

Gene duplication could expand the genome and lead to differential gene functions for optimal adaptability in the evolution of plants (Xu et al., 2012). There are three mechanism contributing to the gene family expansion, i.e., tandem duplication, segmental duplication, and whole-genome duplication (WGD; Xu et al., 2012). WGDs were assumed to have played a major role in the diversification of angiosperms (Soltis et al., 2009). Mesopolyploidization is one category of WGD events, and it has been defined in Brassicaceae (Mandakova et al., 2010). The progenitor diploid genomes of *B. napus* are ancient polyploids (Schmidt et al., 2001), and *B. napus* was formed by allopolyploidy (Chalhoub et al., 2014). Many research studies have revealed that the *Brassica* species has undergone WGD events during their evolution; in addition, several independent lineage-specific WGD events have been identified in Brassicaceae (Rana et al., 2004; Cheng et al., 2014). In this study, the *BnaVOC* superfamily formation was associated with segmental duplications and WGD. In *Arabidopsis* and rice, segmental duplication events were also found in the *VOC* family genes (Mustafiz et al., 2011). The *Arabidopsis* genome contains 22 GLYI genes; therefore, a WGT event would be expected to produce more than 66 GLYI genes in the *B. oleracea* or *B. rapa* genome, ultimately leading to even more GLYI genes in *B. napus*. However, only 34 genes were observed in the *B. napus* genome in the present study. This finding implies that more than 50% of the duplicated GLY I genes were lost after WGT, which might be due to the extensive chromosome reshuffling during the rediploidization after WGT (Cheng et al., 2014). Most likely, 34 GLYI genes were sufficient for *B. napus* during the long natural selection process, and thus, some duplicated GLYI genes did not remain in the *B. napus* genome. For example, certain genes that are homologous to *Arabidopsis* (AT5G41650, AT1G67280, AT2G28420, AT2G32090, AT2G32090, AT5G57040) were detected with less than four copies in *B. napus*; thus, the eleven genes that were not detected might have been lost (Table 2). Similar losses of genes after WGT have also been observed in other gene families in *Brassica* (Yu et al., 2014; Liang et al., 2016). The synteny analysis demonstrated that most *VOC* gene family members are located in well-conserved synteny regions, and *VOC* genes in the A genomes from *B. rapa* and C genomes from *B. oleracea* exhibited a greater homology to *B. napus* than to *A. thaliana*. WGDs play a major role in *Brassica*s, particularly the mesopolyploidization events, which are simultaneously accompanied by extensive chromosomal and genetic diploidization processes (Hohmann et al., 2015). After WGT, extensive genome fractionation, block reshuffling and chromosome reduction accompanied by paleocentromere, which was a descendent of the tPCK subgenomes during the rediploidization process, produced stable diploid species (Schranz et al., 2006; Cheng et al., 2014). All the *VOC* genes in the *Brassica* species have a syntenic relationship with the tPCK chromosome. The characteristics of the *BnaVOC* family duplication patterns and synteny analysis were consistent with the *Brassicaceae* evolutionary history.

### Evolution and phylogenetic analysis of the VOC family genes

An evolutionary pathway for the structural scaffolding of the VOC superfamily has been proposed (Bergdoll et al., 1998), and then, the pathway was modified (Armstrong, 2000). Usually, gene fusion and duplication can create a stronger two-motif pseudosymmetric metallomonomer, which enhances the utility of the dimer. This evolutionary step in the VOC family genes was also observed in *B. napus*. The phylogenetic, evolution and structural analysis results revealed that the BnaVOCs have a closer phylogenetic relationship with a similar structure, and their Ka/Ks ratio confirmed this result (Figures 4, 5, Table 2). Most *BnaVOC*s do not have closer phylogenetic relationships with *B. nigra* than with other *Brassica* species (Figure S4). This result is consistent with the evolutionary history of *Brassica* (Hohmann et al., 2015). The progenitor of the VOC superfamily is assumed to be a mini gene-encoded single βαβββ motif (Armstrong, 2000); thus, the members of *BnaVOC*s in the phylogenetic clade that contains more βαβββ motifs likely resulted from this evolution step. As discussed above, the *BnaVOC* family genes experienced WGD and segmental duplication, these events may have also affected their structural formation in evolution. *BnaHPPD4* is in a single phylogenetic clade, and its structure was different with that of the other *BnaHPPD*s, which likely resulted from certain additional gene duplication-fusion events. Such events can provide four motif monomers as is eventually observed in some of the extradiol dioxygenases and yeast GLYI (Ridderstrom and Mannervik, 1996; Armstrong, 2000).

### Expression profile analysis reveals that the *BnaVOC* genes have diverse expression patterns in normal tissues under dry stress conditions

The *BnaVOC* genes have different expression patterns both in normal tissues and under dry stress conditions. The expression in late stage seeds was obviously higher than that in the early stage seeds, which indicated that the BnaVOCs accumulated during the dehydration progress, which is a feature that is consistent with certain VOCs in other plants (Mulako et al., 2008). High expression of *BnaVOC*s was also observed in the leaf, and the leaves were closely connected to abiotic stress. GLYIs in *Arabidopsis* and rice were highly expressed under abiotic stress conditions (Mustafiz et al., 2011), and GLYIs in rice were reported to function under abiotic stress conditions (Mustafiz et al., 2014). After the dry stress treatment, the *B. napus* seeds showed a higher expression level (Figure 7), and all these results suggested that the BnaVOCs might also function under certain abiotic stress conditions, particularly dry stress. Dsi-1VOC proteins were found in the high oil content *B. napus* lines in our previous study (Gan et al., 2013), and certain VOC proteins could also be induced in seed desiccation (Mulako et al., 2008); additionally, the functional networks of the BnaVOCs and dsi-1VOC proteins show that they are linked with genes that are involved in drought stress or lipid metabolism (Figure S3); therefore, the BnaVOCs are likely involved in lipid formation, and the expression profiles of the *BnaVOC* genes in this study support this assumption. Under the dry stress condition, the high oil content *B. napus* lines showed a higher level of expression of the *BnaVOC* genes than that in the low oil content lines, which might be due to their biological function of tolerance to methylglyoxal and their participation in lipid metabolism.

The glyoxalase system plays major roles in the detoxification of methylglyoxal (MG), and MG is a cytotoxic by-product of the glycolytic pathway by catalyzing the reaction forming S-Dlactoylglutathione (GSH; Singla-Pareek et al., 2003). MG was involved in abiotic stress and hormonal responses in plants (Mustafiz et al., 2014), and GSH affects lipid peroxidation (De La Cruz et al., 2000); all these results suggest that GLYI is important in lipid metabolism in plants under abiotic stress conditions. HPPDs catalyse the reaction from 4-hydroxyphenylpyruvate (HPP) to 2,5-dihydroxyphenylacetate (homogentisate, HG), which is at an intersection of certain important biological processes (Falk et al., 2003; He and Moran, 2011). HG is vital for photosynthesis in plants because it is the precursor to tocopherol and plastoquinone, which are both important for photosynthesis systems (Sakuragi et al., 2006). Under the dry stress condition, more efficient photosynthesis helps the formation of carbohydrate (Alonso et al., 2010), and carbohydrate is interrelated with the oil content in *B. napus* (King et al., 1997). In addition, the displacement of metals from metalloenzymes or metabolites is one of the five mechanisms linked with heavy metal toxicity, which could lead to membrane structural changes (Baker et al., 2006). Fatty acids (FAs) were also connected with the membrane structure and were closely related to heavy metal tolerance and abiotic stress in higher plants (Maksymiec, 1998; Zemanova et al., 2015). These results suggested that the BnaVOCs may affect lipid metabolism and show different expression pattern in high and low oil content *B. napus* lines under dry stress conditions.

## Conclusion

In this study, 38 *BnaVOC* genes were identified and a comprehensive analysis was performed in which the conserved structures in the VOC superfamily were observed; WGD and segmental duplication contributed to the *BnaVOC* gene family duplication in the phylogenetic evolution. The expression profile analysis provided novel insight into the biological function of the BnaVOC protein, and BnaVOCs not only respond to abiotic stress but may also affect lipid metabolism and oil formation.

## Author contributions

YL did the identification of VOC family genes in *B. napus*, duplication pattern analysis and wrote the main manuscript text. NW did multiple alignment of the *BnaVOC* genes and made the phylogenetic analysis. YL, NW, ZC, YM, and BL did the qRT-PCR, collected *VOC* genes information of other species, and did structural analysis and prepared Figures 1–7. HL, NR, and YY prepared for supplementary information. ML designed the experiment. All authors reviewed the manuscript.

### Conflict of interest statement

The authors declare that the research was conducted in the absence of any commercial or financial relationships that could be construed as a potential conflict of interest.

## Supplementary material

### Availability of data and materials

The data set of Figures 1, 5 supporting the results of this article is available in the TreeBase (https://treebase.org/treebase-web/home.html) repository, under the accession URL (http://purl.org/phylo/treebase/phylows/study/TB2:S20148). The genes sequences are available in CNS-Genoscope database (http://www.genoscope.cns.fr/brassicanapus/).

## References

[B1] Allender C. J. King G. J. (2010). Origins of the amphiploid species *Brassica napus* L. investigated by chloroplast and nuclear molecular markers. BMC Plant Biol. 10:54. 10.1186/1471-2229-10-54 20350303 PMC2923528

[B2] Alonso D. M. Bond J. Q. Dumesic J. A. (2010). Catalytic conversion of biomass to biofuels. Green Chem. 12, 1493–1513. 10.1039/c004654j

[B3] Armstrong R. N. (2000). Mechanistic diversity in a metalloenzyme superfamily. Biochemistry 39, 13625–13632. 10.1021/bi001814v 11076500

[B4] Bailey T. L. Boden M. Buske F. A. Frith M. Grant C. E. Clementi L.. (2009). MEME SUITE: tools for motif discovery and searching. Nucleic Acids Res. 37, W202–W208. 10.1093/nar/gkp335 19458158 PMC2703892

[B5] Baker A. Graham I. A. Holdsworth M. Smith S. M. Theodoulou F. L. (2006). Chewing the fat: beta-oxidation in signalling and development. Trends Plant Sci. 11, 124–132. 10.1016/j.tplants.2006.01.005 16490379

[B6] Beck E. H. Fettig S. Knake C. Hartig K. Bhattarai T. (2007). Specific and unspecific responses of plants to cold and drought stress. J. Biosci. 32, 501–510. 10.1007/s12038-007-0049-5 17536169

[B7] Bergdoll M. Eltis L. D. Cameron A. D. Dumas P. Bolin J. T. (1998). All in the family: structural and evolutionary relationships among three modular proteins with diverse functions and variable assembly. Protein Sci. 7, 1661–1670. 10.1002/pro.5560070801 10082363 PMC2144073

[B8] Bernat B. A. Laughlin L. T. Armstrong R. N. (1997). Fosfomycin resistance protein (FosA) is a manganese metalloglutathione transferase related to glyoxalase I and the extradiol dioxygenases. Biochemistry 36, 3050–3055. 10.1021/bi963172a 9115979

[B9] Bodén M. Hawkins J. (2005). Prediction of subcellular localization using sequence-biased recurrent networks. Bioinformatics 21, 2279–2286. 10.1093/bioinformatics/bti372 15746276

[B10] Brownlee J. He P. Moran G. R. Harrison D. H. (2008). Two roads diverged: the structure of hydroxymandelate synthase from Amycolatopsis orientalis in complex with 4-hydroxymandelate. Biochemistry 47, 2002–2013. 10.1021/bi701438r 18215022

[B11] Buchan D. W. Minneci F. Nugent T. C. Bryson K. Jones D. T. (2013). Scalable web services for the PSIPRED Protein Analysis Workbench. Nucleic Acids Res. 41, W349–W357. 10.1093/nar/gkt381 23748958 PMC3692098

[B12] Chalhoub B. Denoeud F. Liu S. Parkin I. A. Tang H. Wang X.. (2014). Plant genetics. Early allopolyploid evolution in the post-Neolithic *Brassica napus* oilseed genome. Science 345, 950–953. 10.1126/science.1253435 25146293

[B13] Cheng F. Wu J. Wang X. (2014). Genome triplication drove the diversification of Brassica plants. Hortic. Res. 1, 14024. 10.1038/hortres.2014.24 26504539 PMC4596316

[B14] De La Cruz J. P. Quintero L. Villalobos M. A. Sanchez de la Cuesta F. (2000). Lipid peroxidation and glutathione system in hyperlipemic rabbits: influence of olive oil administration. Biochim. Biophys. Acta 1485, 36–44. 10.1016/S1388-1981(00)00027-5 10802247

[B15] Emanuelsson O. Brunak S. von Heijne G. Nielsen H. (2007). Locating proteins in the cell using TargetP, SignalP and related tools. Nat. Protoc. 2, 953–971. 10.1038/nprot.2007.131 17446895

[B16] Falk J. Andersen G. Kernebeck B. Krupinska K. (2003). Constitutive overexpression of barley 4-hydroxyphenylpyruvate dioxygenase in tobacco results in elevation of the vitamin E content in seeds but not in leaves. FEBS Lett. 540, 35–40. 10.1016/S0014-5793(03)00166-2 12681479

[B17] Falk J. Krauss N. Dahnhardt D. Krupinska K. (2002). The senescence associated gene of barley encoding 4-hydroxyphenylpyruvate dioxygenase is expressed during oxidative stress. J. Plant Physiol. 159, 1245–1253. 10.1078/0176-1617-00804

[B18] Finn R. D. Mistry J. Tate J. Coggill P. Heger A. Pollington J. E.. (2010). The Pfam protein families database. Nucleic Acids Res. 38, D211–D222. 10.1093/nar/gkp985 19920124 PMC2808889

[B19] Gan L. Zhang C. Y. Wang X. D. Wang H. Long Y. Yin Y. T.. (2013). Proteomic and comparative genomic analysis of two *Brassica napus* lines differing in oil content. J. Proteome Res. 12, 4965–4978. 10.1021/pr4005635 24053668

[B20] Gasteiger E. Gattiker A. Hoogland C. Ivanyi I. Appel R. D. Bairoch A. (2003). ExPASy: the proteomics server for in-depth protein knowledge and analysis. Nucleic Acids Res. 31, 3784–3788. 10.1093/nar/gkg563 12824418 PMC168970

[B21] Gerlt J. A. Babbitt P. C. (2001). Divergent evolution of enzymatic function: mechanistically diverse superfamilies and functionally distinct suprafamilies. Annu. Rev. Biochem. 70, 209–246. 10.1146/annurev.biochem.70.1.209 11395407

[B22] He P. Moran G. R. (2011). Structural and mechanistic comparisons of the metal-binding members of the vicinal oxygen chelate (VOC) superfamily. J. Inorg. Biochem. 105, 1259–1272. 10.1016/j.jinorgbio.2011.06.006 21820381

[B23] Higgins D. G. Sharp P. M. (1988). CLUSTAL: a package for performing multiple sequence alignment on a microcomputer. Gene 73, 237–244. 10.1016/0378-1119(88)90330-7 3243435

[B24] Hohmann N. Wolf E. M. Lysak M. A. Koch M. A. (2015). A Time-calibrated road map of Brassicaceae species radiation and evolutionary history. Plant Cell 27, 2770–2784. 10.1105/tpc.15.00482 26410304 PMC4682323

[B25] Hu B. Jin J. Guo A. Y. Zhang H. Luo J. Gao G. (2015). GSDS 2.0: an upgraded gene feature visualization server. Bioinformatics 31, 1296–1297. 10.1093/bioinformatics/btu817 25504850 PMC4393523

[B26] Jones D. T. (1999). Protein secondary structure prediction based on position-specific scoring matrices. J. Mol. Biol. 292, 195–202. 10.1006/jmbi.1999.3091 10493868

[B27] Kagale S. Divi U. K. Krochko J. E. Keller W. A. Krishna P. (2007). Brassinosteroid confers tolerance in *Arabidopsis thaliana* and *Brassica napus* to a range of abiotic stresses. Planta 225, 353–364. 10.1007/s00425-006-0361-6 16906434

[B28] Kelley L. A. Sternberg M. J. E. (2009). Protein structure prediction on the Web: a case study using the Phyre server. Nat. Protoc. 4, 363–371. 10.1038/nprot.2009.2 19247286

[B29] King S. P. Lunn J. E. Furbank R. T. (1997). Carbohydrate content and enzyme metabolism in developing canola siliques. Plant Physiol. 114, 153–160. 10.1104/pp.114.1.153 12223695 PMC158289

[B30] Letunic I. Copley R. R. Schmidt S. Ciccarelli F. D. Doerks T. Schultz J.. (2004). SMART 4.0: towards genomic data integration. Nucleic Acids Res. 32, D142–D144. 10.1093/nar/gkh088 14681379 PMC308822

[B31] Li W. Liu B. Yu L. Feng D. Wang H. Wang J. (2009). Phylogenetic analysis, structural evolution and functional divergence of the 12-oxo-phytodienoate acid reductase gene family in plants. BMC Evol. Biol. 9:90. 10.1186/1471-2148-9-90 19416520 PMC2688005

[B32] Liang Y. Xiong Z. Zheng J. Xu D. Zhu Z. Xiang J.. (2016). Genome-wide identification, structural analysis and new insights into late embryogenesis abundant (LEA) gene family formation pattern in *Brassica napus*. Sci. Rep. 6:24265. 10.1038/srep24265 27072743 PMC4829847

[B33] Liu S. Liu Y. Yang X. Tong C. Edwards D. Parkin I. A.. (2014). The *Brassica oleracea* genome reveals the asymmetrical evolution of polyploid genomes. Nat. Commun. 5:3930. 10.1038/ncomms4930 24852848 PMC4279128

[B34] Maksymiec W. (1998). Effect of copper on cellular processes in higher plants. Photosynthetica 34, 321–342. 10.1023/A:1006818815528

[B35] Mandakova T. Joly S. Krzywinski M. Mummenhoff K. Lysak M. A. (2010). Fast diploidization in close mesopolyploid relatives of Arabidopsis. Plant Cell 22, 2277–2290. 10.1105/tpc.110.074526 20639445 PMC2929090

[B36] Marchler-Bauer A. Derbyshire M. K. Gonzales N. R. Lu S. Chitsaz F. Geer L. Y.. (2015). CDD: NCBI's conserved domain database. Nucleic Acids Res 43, D222–D226. 10.1093/nar/gku1221 25414356 PMC4383992

[B37] McCarthy A. A. Baker H. M. Shewry S. C. Patchett M. L. Baker E. N. (2001). Crystal structure of methylmalonyl-coenzyme A epimerase from *P. shermanii*: a novel enzymatic function on an ancient metal binding scaffold. Structure 9, 637–646. 10.1016/s0969-2126(01)00622-0 11470438

[B38] Mulako I. Farrant J. M. Collett H. Illing N. (2008). Expression of *Xhdsi-1*^VOC^, a novel member of the vicinal oxygen chelate (VOC) metalloenzyme superfamily, is up-regulated in leaves and roots during desiccation in the resurrection plant *Xerophyta humilis* (Bak) Dur and Schinz. J. Exp. Bot. 59, 3885–3901. 10.1093/jxb/ern226 18791196 PMC2576639

[B39] Mustafiz A. Ghosh A. Tripathi A. K. Kaur C. Ganguly A. K. Bhavesh N. S.. (2014). A unique Ni^2+^-dependent and methylglyoxal-inducible rice glyoxalase I possesses a single active site and functions in abiotic stress response. Plant J. 78, 951–963. 10.1111/tpj.12521 24661284

[B40] Mustafiz A. Singh A. K. Pareek A. Sopory S. K. Singla-Pareek S. L. (2011). Genome-wide analysis of rice and Arabidopsis identifies two glyoxalase genes that are highly expressed in abiotic stresses. Funct. Integr. Genomics 11, 293–305. 10.1007/s10142-010-0203-2 21213008

[B41] Nekrutenko A. Makova K. D. Li W.-H. (2002). The KA/KS ratio test for assessing the protein-coding potential of genomic regions: an empirical and simulation study. Genome Res. 12, 198–202. 10.1101/gr.200901 11779845 PMC155263

[B42] Ostergaard L. King G. J. (2008). Standardized gene nomenclature for the Brassica genus. Plant Methods 4:10. 10.1186/1746-4811-4-10 18492252 PMC2408569

[B43] Rana D. van den Boogaart T. O'Neill C. M. Hynes L. Bent E. Macpherson L.. (2004). Conservation of the microstructure of genome segments in *Brassica napus* and its diploid relatives. Plant J. 40, 725–733. 10.1111/j.1365-313X.2004.02244.x 15546355

[B44] Reddy V. S. Sopory S. K. (1999). Glyoxalase I from Brassica juncea: molecular cloning, regulation and its over-expression confer tolerance in transgenic tobacco under stress. Plant J. 17, 385–395. 10.1046/j.1365-313X.1999.00390.x 10205896

[B45] Ridderstrom M. Mannervik B. (1996). The primary structure of monomeric yeast glyoxalase I indicates a gene duplication resulting in two similar segments homologous with the subunit of dimeric human glyoxalase I. Biochem. J. 316 (Pt 3), 1005–1006. 10.1042/bj3161005 8670139 PMC1217405

[B46] Saint-Jean A. P. Phillips K. R. Creighton D. J. Stone M. J. (1998). Active monomeric and dimeric forms of *Pseudomonas putida* glyoxalase I: evidence for 3D domain swapping. Biochemistry 37, 10345–10353. 10.1021/bi980868q 9671502

[B47] Sakuragi Y. Maeda H. DellaPenna D. Bryant D. A. (2006). α-Tocopherol plays a role in photosynthesis and macronutrient homeostasis of the cyanobacterium *Synechocystis* sp. PCC 6803 that is independent of its antioxidant function. Plant Physiol. 141, 508–521. 10.1104/pp.105.074765 16565298 PMC1475434

[B48] Schlueter J. A. Vaslenko-Sanders I. F. Deshpande S. Yi J. Siegfried M. Roe B. A. (2007). The FAD2 gene family of soybean: insights into the structural and functional divergence of a paleoplyploid genome. Crop Sci. 47, S14–S26. 10.2135/cropsci2006.06.0382tpg

[B49] Schmidt R. Acarkan A. Boivin K. (2001). Comparative structural genomics in the Brassicaceae family. Plant Physiol. Biochem. 39, 253–262. 10.1016/S0981-9428(01)01239-6

[B50] Schranz M. E. Lysak M. A. Mitchell-Olds T. (2006). The ABC's of comparative genomics in the Brassicaceae: building blocks of crucifer genomes. Trends Plant Sci. 11, 535–542. 10.1016/j.tplants.2006.09.002 17029932

[B51] Seki M. Narusaka M. Abe H. Kasuga M. Yamaguchi-Shinozaki K. Carninci P.. (2001). Monitoring the expression pattern of 1300 Arabidopsis genes under drought and cold stresses by using a full-length cDNA microarray. Plant Cell 13, 61–72. 10.1105/tpc.13.1.61 11158529 PMC102214

[B52] Singla-Pareek S. L. Reddy M. K. Sopory S. K. (2003). Genetic engineering of the glyoxalase pathway in tobacco leads to enhanced salinity tolerance. Proc. Natl. Acad. Sci. U.S.A. 100, 14672–14677. 10.1073/pnas.2034667100 14638937 PMC299757

[B53] Soltis D. E. Albert V. A. Leebens-Mack J. Bell C. D. Paterson A. H. Zheng C.. (2009). Polyploidy and angiosperm diversification. Am. J. Bot. 96, 336–348. 10.3732/ajb.0800079 21628192

[B54] Tamura K. Stecher G. Peterson D. Filipski A. Kumar S. (2013). MEGA6: Molecular Evolutionary Genetics Analysis version 6.0. Mol. Biol. Evol. 30, 2725–2729. 10.1093/molbev/mst197 24132122 PMC3840312

[B55] Thornalley P. J. (1990). The glyoxalase system: new developments towards functional characterization of a metabolic pathway fundamental to biological life. Biochem. J. 269, 1–11. 10.1042/bj2690001 2198020 PMC1131522

[B56] Thornalley P. J. (2003). Glyoxalase I–structure, function and a critical role in the enzymatic defence against glycation. Biochem. Soc. Trans. 31(Pt 6), 1343–1348. 10.1042/bst0311343 14641060

[B57] Wang X. Wang H. Wang J. Sun R. Wu J. Liu S.. (2011). The genome of the mesopolyploid crop species *Brassica rapa*. Nat. Genet. 43, 1035–1039. 10.1038/ng.919 21873998

[B58] Xiong L. Zhu J. K. (2002). Molecular and genetic aspects of plant responses to osmotic stress. Plant Cell Environ. 25, 131–139. 10.1046/j.1365-3040.2002.00782.x 11841658

[B59] Xu G. Guo C. Shan H. Kong H. (2012). Divergence of duplicate genes in exon-intron structure. Proc. Natl. Acad. Sci. U.S.A. 109, 1187–1192. 10.1073/pnas.1109047109 22232673 PMC3268293

[B60] Yang Z. (2007). PAML 4: phylogenetic analysis by maximum likelihood. Mol. Biol. Evol. 24, 1586–1591. 10.1093/molbev/msm088 17483113

[B61] Yu J. Y. Tehrim S. Zhang F. Q. Tong C. B. Huang J. Y. Cheng X. H.. (2014). Genome-wide comparative analysis of NBS-encoding genes between Brassica species and *Arabidopsis thaliana*. BMC Genomics 15:3. 10.1186/1471-2164-15-3 24383931 PMC4008172

[B62] Zemanova V. Pavlik M. Kyjakova P. Pavlikova D. (2015). Fatty acid profiles of ecotypes of hyperaccumulator *Noccaea caerulescens* growing under cadmium stress. J. Plant Physiol. 180, 27–34. 10.1016/j.jplph.2015.02.012 25886397

