# Supplementary material for: Whole-Genome Identification and Expression Pattern of the Vicinal Oxygen Chelate Family in Rapeseed (Brassica napus L.)
Source: Front Plant Sci. 2017 May 9;8:745. doi: 10.3389/fpls.2017.00745 (PMC5422514; doi:10.3389/fpls.2017.00745)
Supplement: Supplementary file 5 [file Image1.pdf]

Whole-genome identification and expression pattern of the vicinal oxygen chelate family in Rapeseeed (*Brassica napus* L .)

Yu Liang[1, 2], Neng Wan[1], Zao Cheng[1], Yufeng Mo[1], Baolin Liu[1], Hui Liu[1], Nadia Raboanatahiry [1], Yongtai Yin[1], Maoteng Li[1, 2]*

[1] Department of Biotechnology, College of Life Science and Technology, Huazhong University of Science and Technology, Wuhan, China, 430074.

[2] Hubei Collaborative Innovation Center for the Characteristic Resources Exploitation of Dabie Mountains, Huanggang Normal University, Huanggang 438000, China

*correspondence author: E-mail: limaoteng426@mail.hust.edu.cn

Supplementary Information:

Image legends

Image S1. Motif patterns of different *BnaVOC* families and WebLogo plot of consensus motifs in each *BnaVOC* gene family. The *B. napus* VOC proteins were selected for alignment, and VOC motifs are shown as motif 2 (light blue box). The Pfam codes of the VOC motifs of each family are shown. The lengths of the *BnaVOC* genes can be estimated using the scale at the bottom.

Image S1

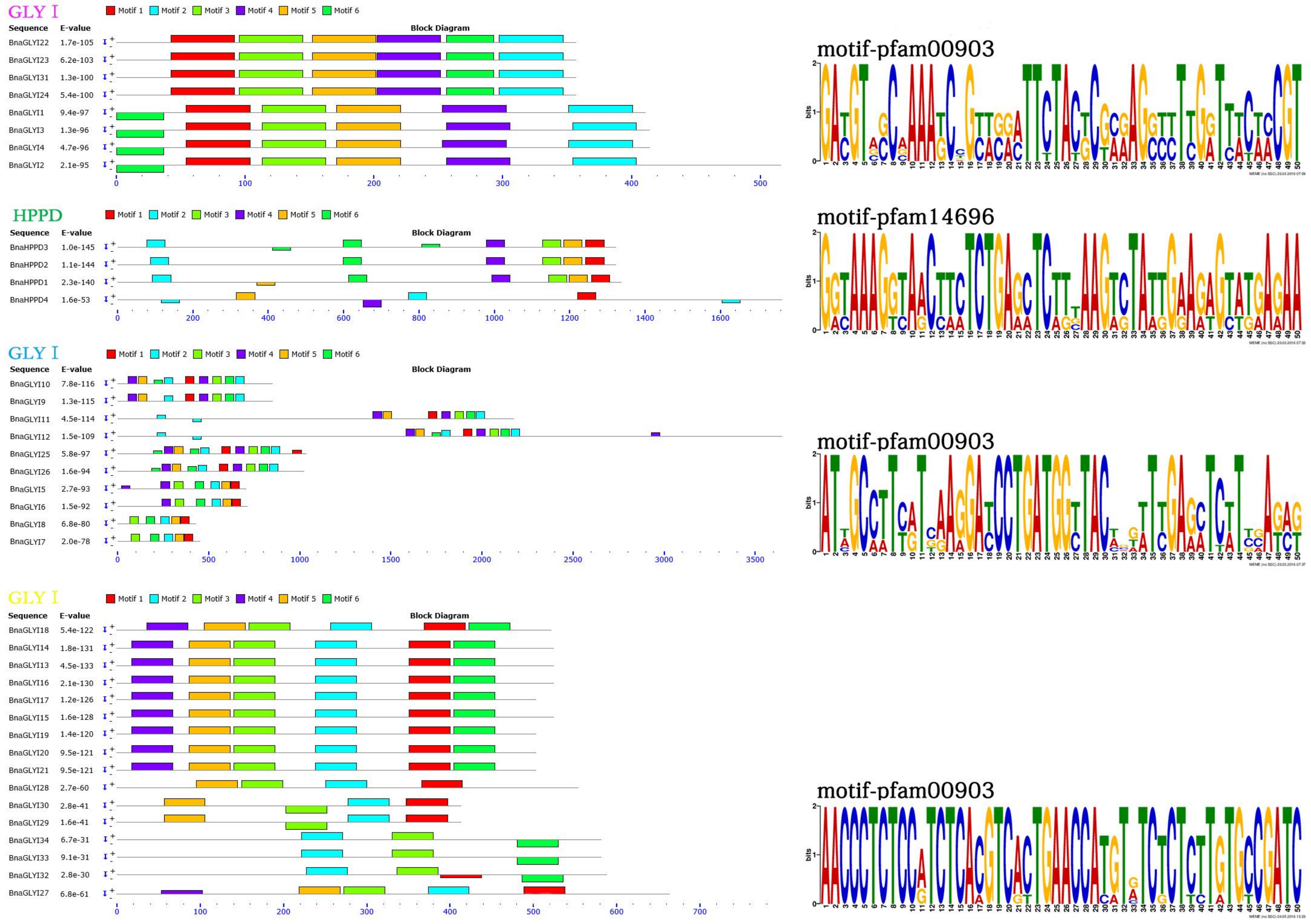

GLY I
Motif 1 Motif 2 Motif 3 Motif 4 Motif 5 Motif 6
Sequence E-value Block Diagram
BnaGLYI22 1.7e-105
BnaGLYI23 6.2e-103
BnaGLYI31 1.3e-100
BnaGLYI24 5.4e-100
BnaGLYI1 9.4e-97
BnaGLYI3 1.3e-96
BnaGLYI4 4.7e-96
BnaGLYI2 2.1e-95
0 100 200 300 400 500
motif-pfam00903
HPPD
Motif 1 Motif 2 Motif 3 Motif 4 Motif 5 Motif 6
Sequence E-value Block Diagram
BnaHPPD3 1.0e-145
BnaHPPD2 1.1e-144
BnaHPPD1 2.3e-140
BnaHPPD4 1.6e-53
0 200 400 600 800 1000 1200 1400 1600
motif-pfam14696
GLY I
Motif 1 Motif 2 Motif 3 Motif 4 Motif 5 Motif 6
Sequence E-value Block Diagram
BnaGLYI10 7.8e-116
BnaGLYI9 1.3e-115
BnaGLYI11 4.5e-114
BnaGLYI12 1.5e-109
BnaGLYI25 5.8e-97
BnaGLYI26 1.6e-94
BnaGLYI5 2.7e-93
BnaGLYI6 1.5e-92
BnaGLYI8 6.8e-80
BnaGLYI7 2.0e-78
0 500 1000 1500 2000 2500 3000 3500
motif-pfam00903
GLY I
Motif 1 Motif 2 Motif 3 Motif 4 Motif 5 Motif 6
Sequence E-value Block Diagram
BnaGLYI18 5.4e-122
BnaGLYI14 1.8e-131
BnaGLYI13 4.5e-133
BnaGLYI16 2.1e-130
BnaGLYI17 1.2e-126
BnaGLYI15 1.6e-128
BnaGLYI19 1.4e-120
BnaGLYI20 9.5e-121
BnaGLYI21 9.5e-121
BnaGLYI28 2.7e-60
BnaGLYI30 2.8e-41
BnaGLYI29 1.6e-41
BnaGLYI34 6.7e-31
BnaGLYI33 9.1e-31
BnaGLYI32 2.8e-30
BnaGLYI27 6.8e-61
0 100 200 300 400 500 600 700
motif-pfam00903
bits
