# Supplementary material for: Whole-Genome Identification and Expression Pattern of the Vicinal Oxygen Chelate Family in Rapeseed (Brassica napus L.)
Source: Front Plant Sci. 2017 May 9;8:745. doi: 10.3389/fpls.2017.00745 (PMC5422514; doi:10.3389/fpls.2017.00745)
Supplement: Supplementary file 6 [file Image2.pdf]

Whole-genome identification and expression pattern of the vicinal oxygen chelate family in Rapeseeed (*Brassica napus* L .)

Yu Liang[1, 2], Neng Wan[1], Zao Cheng[1], Yufeng Mo[1], Baolin Liu[1], Hui Liu[1], Nadia Raboanatahiry [1], Yongtai Yin[1], Maoteng Li[1, 2]*

[1] Department of Biotechnology, College of Life Science and Technology, Huazhong University of Science and Technology, Wuhan, China, 430074.

[2] Hubei Collaborative Innovation Center for the Characteristic Resources Exploitation of Dabie Mountains, Huanggang Normal University, Huanggang 438000, China

*correspondence author: E-mail: limaoteng426@mail.hust.edu.cn

Supplementary Information:

Image legends

Image S2. Predicted secondary structure of BnaGLYI. BnaGLYI1 was an example to show that results from GOR4 prediction and PSIPRED is relatively the same. A: Results from GOR4 prediction analysis. B: Results from PSIPRED prediction analysis.

Image S2

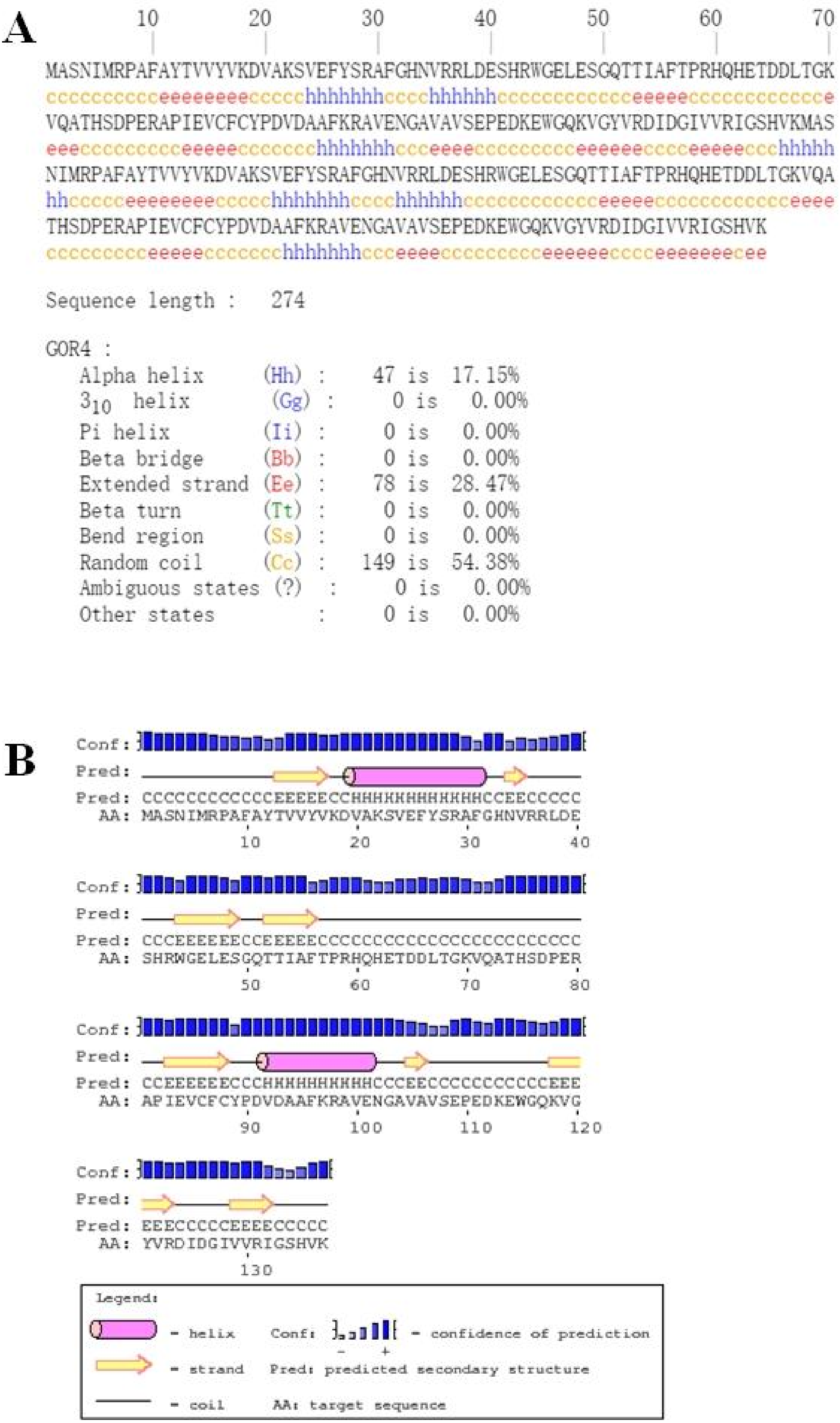
A
10 20 30 40 50 60 70
MASNIMRPAFAYTVVYVKDVAKSVEFYSRAFGHNVRRLDESHRWGELESGQTTIAFTPRHQHETDDLTGK
cccccccccceeeeeeeeccccchhhhhhhcccchhhhhhcccccccccccceeeeecccccccccccce
VQATHSDPERAPIEVCFCYPDVDAAFKRAVENGAVAVSEPEDKEWGQKVGYVRDIDGIVVRIGSHVKMAS
eeeccccccccceeeeeccccccchhhhhhhccceeeeccccccccceeeeeecccceeeeeccchhhhh
NIMRPAFAYTVVYVKDVAKSVEFYSRAFGHNVRRLDESHRWGELESGQTTIAFTPRHQHETDDLTGKVQA
hhccccceeeeeeeeccccchhhhhhhcccchhhhhhcccccccccccceeeeecccccccccccceeee
THSDPERAPIEVCFCYPDVDAAFKRAVENGAVAVSEPEDKEWGQKVGYVRDIDGIVVRIGSHVK
ccccccccceeeeeccccccchhhhhhhccceeeeccccccccceeeeeecccceeeeeeecee
Sequence length : 274
GOR4 :
Alpha helix (Hh) : 47 is 17.15%
3₁₀ helix (Gg) : 0 is 0.00%
Pi helix (Ii) : 0 is 0.00%
Beta bridge (Bb) : 0 is 0.00%
Extended strand (Ee) : 78 is 28.47%
Beta turn (Tt) : 0 is 0.00%
Bend region (Ss) : 0 is 0.00%
Random coil (Cc) : 149 is 54.38%
Ambiguous states (?) : 0 is 0.00%
Other states : 0 is 0.00%
B
Conf:
Pred:
Pred: CCCCCCCCCCCCEEEEECCHHHHHHHHHHHHCCEECCCCC
AA: MASNIMRPAFAYTVVYVKDVAKSVEFYSRAFGHNVRRLDE
10 20 30 40
Conf:
Pred:
Pred: CCCEEEEEECCEEEEECCCCCCCCCCCCCCCCCCCCCCCC
AA: SHRWGELESGQTTIAFTPRHQHETDDLTGKVQATHSDPER
50 60 70 80
Conf:
Pred:
Pred: CCEEEEEEECCCHHHHHHHHHCCCEECCCCCCCCCCCEEE
AA: APIEVCFCYPDVDAAFKRAVENGAVAVSEPEDKEWGQKVG
90 100 110 120
Conf:
Pred:
Pred: EEECCCCCEEEECCCCC
AA: YVRDIDGIVVRIGSHVK
130
Legend:
- helix
Conf:
- confidence of prediction
- +
- strand
Pred: predicted secondary structure
- coil
AA: target sequence
