# Supplementary material for: Whole-Genome Identification and Expression Pattern of the Vicinal Oxygen Chelate Family in Rapeseed (Brassica napus L.)
Source: Front Plant Sci. 2017 May 9;8:745. doi: 10.3389/fpls.2017.00745 (PMC5422514; doi:10.3389/fpls.2017.00745)
Supplement: Supplementary file 7 [file Image3.pdf]

Whole-genome identification and expression pattern of the vicinal oxygen chelate family in Rapeseeed (*Brassica napus* L .)

Yu Liang[1, 2], Neng Wan[1], Zao Cheng[1], Yufeng Mo[1], Baolin Liu[1], Hui Liu[1], Nadia Raboanatahiry [1], Yongtai Yin[1], Maoteng Li[1, 2]*

[1] Department of Biotechnology, College of Life Science and Technology, Huazhong University of Science and Technology, Wuhan, China, 430074.

[2] Hubei Collaborative Innovation Center for the Characteristic Resources Exploitation of Dabie Mountains, Huanggang Normal University, Huanggang 438000, China

*correspondence author: E-mail: limaoteng426@mail.hust.edu.cn

Supplementary Information:

Image legends

Image S3. Predicted functional network of VOC proteins. The VOC amino acid sequences from *B. napus* and *Arabidopsis* were used to be analyzed by STRING. A: Result from BnaVOC proteins with possible functional network. B: Result from dsi-1^{VOC} proteins in *Arabidopsis* with possible functional network.

Image S3

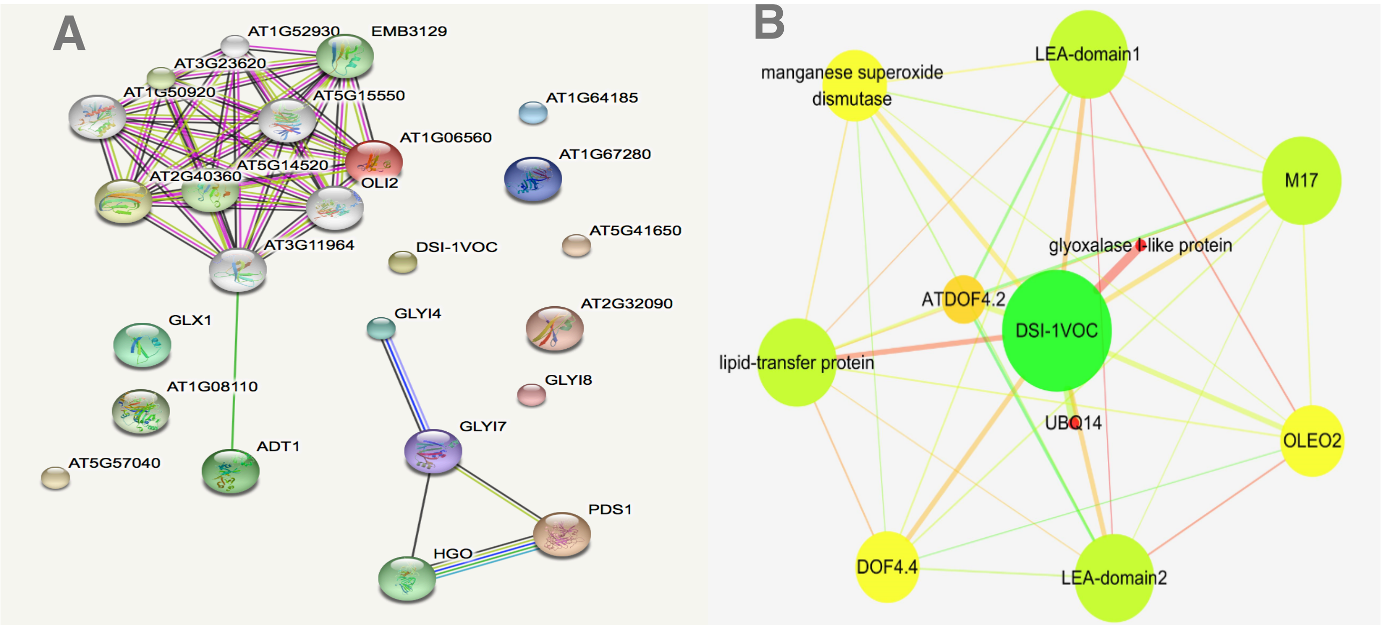
A
AT1G52930
EMB3129
AT3G23620
AT1G50920
AT5G15550
AT1G64185
AT1G06560
AT1G67280
AT5G14520
AT2G40360
OLI2
AT5G41650
AT3G11964
DSI-1VOC
AT2G32090
GLYI4
GLX1
AT1G08110
GLYI8
GLYI7
ADT1
AT5G57040
PDS1
HGO
B
LEA-domain1
manganese superoxide
dismutase
M17
glyoxalase I-like protein
ATDOF4.2
DSI-1VOC
lipid-transfer protein
UBQ14
OLEO2
DOF4.4
LEA-domain2
