# Supplementary material for: Whole-Genome Identification and Expression Pattern of the Vicinal Oxygen Chelate Family in Rapeseed (Brassica napus L.)
Source: Front Plant Sci. 2017 May 9;8:745. doi: 10.3389/fpls.2017.00745 (PMC5422514; doi:10.3389/fpls.2017.00745)
Supplement: Supplementary file 8 [file Image4.pdf]

Whole-genome identification and expression pattern of the vicinal oxygen chelate family in Rapeseeed (*Brassica napus* L .)

Yu Liang[1, 2], Neng Wan[1], Zao Cheng[1], Yufeng Mo[1], Baolin Liu[1], Hui Liu[1], Nadia Raboanatahiry [1], Yongtai Yin[1], Maoteng Li[1, 2]*

[1] Department of Biotechnology, College of Life Science and Technology, Huazhong University of Science and Technology, Wuhan, China, 430074.

[2] Hubei Collaborative Innovation Center for the Characteristic Resources Exploitation of Dabie Mountains, Huanggang Normal University, Huanggang 438000, China

*correspondence author: E-mail: limaoteng426@mail.hust.edu.cn

Supplementary Information:

Image legends

Image S4. Phylogenetic analysis of the *Brassica* species. *B. napus*, *B. rapa*, *B. oleracea* and *B. nigra* VOC genes were used for analysis. Different clades are distinguished by different colors. The unrooted tree was generated by using ClustalW in MEGA with the full-length amino acid sequences of the VOC proteins.

ImageS4

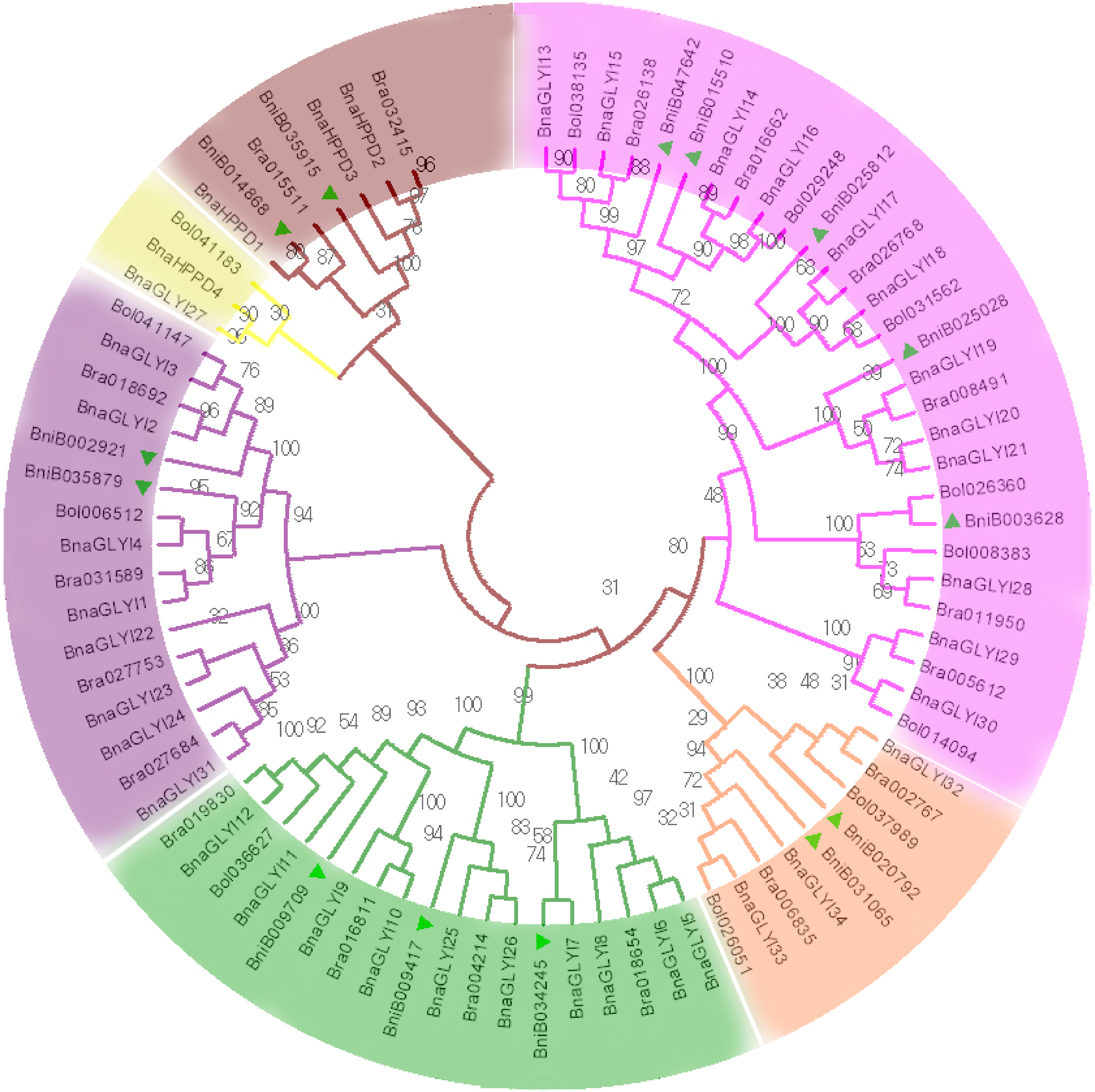
BnaGLYI13
Bol038135
BnaGLYI15
Bra026138
BniB047642
BniB015510
BnaGLYI14
Bra016662
BnaGLYI16
Bol029248
BniB025812
BnaGLYI17
Bra026768
BnaGLYI18
Bol031562
BniB025028
BnaGLYI19
Bra008491
BnaGLYI20
BnaGLYI21
Bol026360
BniB003628
Bol008383
BnaGLYI28
Bra011950
BnaGLYI29
Bra005612
BnaGLYI30
Bol014094
BnaGLYI32
Bra002767
Bol037989
BniB020792
BniB031065
BnaGLYI34
Bra006835
BnaGLYI33
Bol026051
BnaGLYI5
BnaGLYI6
Bra018654
BnaGLYI8
BnaGLYI7
BniB034245
BnaGLYI26
Bra004214
BnaGLYI25
BniB009417
BnaGLYI10
Bra016811
BnaGLYI9
BniB009709
BnaGLYI11
Bol036627
BnaGLYI12
Bra019830
BnaGLYI31
Bra027684
BnaGLYI24
BnaGLYI23
Bra027753
BnaGLYI22
BnaGLYI1
Bra031589
BnaGLYI4
Bol006512
BniB035879
BniB002921
BnaGLYI2
Bra018692
BnaGLYI3
Bol041147
BnaGLYI27
BnaHPPD4
Bol041183
BnaHPPD1
BniB014868
Bra015511
BniB035915
BnaHPPD3
BnaHPPD2
Bra032415
